# Depletion of the RNA‐Editing Enzyme ADAR1 Invigorates the Antitumor Immunity of NK Cells

**DOI:** 10.1002/advs.202517216

**Published:** 2026-01-20

**Authors:** Shuhan Chen, Di Lu, Rukang Liang, Weikeng Tan, Erming Zhao, Sihuang Wu, Xintong Li, Yulong Song, Miaojian Wan, Xiaoyuan Xie, Qi Zhang, Qiuli Liu

**Affiliations:** ^1^ Biotherapy Center The Third Affiliated Hospital Sun Yat‐sen University Guangzhou China; ^2^ Vaccine Research Institute of Sun Yat‐Sen University Guangzhou China; ^3^ State Key Laboratory of Respiratory Disease The Affiliated Panyu Central Hospital of Guangzhou Medical University State Key Laboratory of Respiratory Disease GMU‐GIBH Joint School of Life Sciences, Guangdong Provincial Key Laboratory of Protein Modification and Degradation/Disease, The Guangdong‐Hong Kong‐Macau Joint Laboratory for Cell Fate Regulation and Diseases Guangzhou Medical University Guangzhou Guangdong 511436 China; ^4^ Department of Dermatology and Cosmetic Surgery The Third Affiliated Hospital of Sun Yat‐sen University Guangzhou China; ^5^ Cell‐Gene Therapy Center (CGTC), Institute for Frontier Interdisciplinary Research in Health Sciences and Technology Sun Yat‑sen University Guangzhou China

**Keywords:** ADAR1, anti‐tumor immunity, CD38, natural killer cells

## Abstract

Functional exhaustion and inefficient tumor infiltration rates limit the effectiveness of natural killer (NK) cell‐based cancer immunotherapy. Although the role of adenosine deaminase acting on RNA 1 (ADAR1) in immune cells and tumorigenesis is gradually gaining attention, its role in NK cells is elusive. In this study, we find that ADAR1 expression level is increased in peripheral blood (PB)‐NK cells from patients with melanoma, concurrently exhibiting impaired tumor killing capacity. ADAR1‐knockdown NK cells show enhanced antitumor activity in vitro and in vivo. Compared with *ADAR1* ^flox/flox^ wild‐type (*ADAR1* WT) mice, *Ncr1^iCre/+;^ADAR1* ^flox/flox^ conditional knockout (*ADAR1* cKO) mice present improved tumor control and increased NK cell infiltration. RNA sequencing (RNA‐seq) analysis reveals that ADAR1 knockdown in NK cells (NK shADAR1)  exhibit an activation phenotype with high cell migration potential. A greater level of tumor infiltration by NK shADAR1 cells is verified in 3D Matrigel‐spheroid experiments. Mechanistically, ADAR1 deficiency in NK cells is accompanied by CD38 expression decline via affecting its mRNA stability, resulting in increased cell mobility, proliferation, and tumor killing capacity. Our findings validate ADAR1 as an emerging therapeutic target for enhanced NK cell immunotherapy.

## Graphical abstract

ADAR1 is upregulated in NK cells from melanoma patients, impairing their function. Its loss enhances NK cell tumor infiltration and cytotoxicity in vitro and in vivo. Mechanistically, ADAR1 deficiency destabilizes CD38 mRNA to reduce its expression, thereby increasing NK cell mobility and killing, which nominates it as a therapeutic target for NK cell immunotherapy.

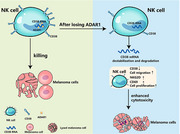

**Abbreviations**
ADAR1: Adenosine deaminase acting on RNA 1
ADO: Adenosine
CFSE: Carboxyfluorescein succinimidyl ester
cKO: Conditional knockout
CTLA‐4: Cytotoxic T lymphocyte‐associated antigen‐4
DEGs: Differentially expressed genes
dsRNA: Double‐stranded RNA
FACS: Fluorescence activated cell sorter
FasL: Fas ligand;
GEO: Gene Expression Omnibus
GO: Gene Ontology
GSEA: Gene Set Enrichment Analysis
HE: Hematoxylin and eosin
i.h.: Hypodermic injected
i.v.: Intravenous injection
ISGs: IFN‐stimulated genes;
KEGG: Kyoto Encyclopedia of Genes and Genomes
LDH: Lactate dehydrogenase
MDA5: Melanoma differentiation‐associated gene 5
MFI: Mean fluorescence intensity
NAD+: Nicotinamide adenine dinucleotide
NAM: Nicotinamide
NK cells: Natural killer cells
NMN: Nicotinamide Mononucleotide
PBMCs: Peripheral blood mononuclear cells;
PD‐1: Programmed cell death protein 1
PEI: Polyethylenimine
PI: Propidium iodide
qRT‐PCR: Quantitative real time‐polymerase chain reaction
RIG‐I: Retinoic acid inducible gene‐I
TME: Tumor microenvironment
TRAIL: TNF‐related apoptosis‐inducing ligand;

## 1 Introduction

Melanoma is caused primarily by the abnormal growth of melanocytes due to excessive ultraviolet radiation and can be classified as cutaneous melanoma (CM), uveal melanoma (UM), mucosal melanoma (MM), or acral melanoma (AM) depending on the location of the primary tumor. Among these subtypes, CM has the highest incidence [1]. The CM incidence rate has doubled in the last 30 years [2]. Malignant melanoma is a leading cause of skin cancer death; it can spread to other parts of the body, resulting in more than 9000 deaths each year (https://www.cdc.gov/vitalsigns/melanoma/). The most effective therapeutic strategy for melanoma is surgical resection, whereas radiotherapy and chemotherapy have traditionally been used for unresectable metastatic melanoma. However, these two therapies have many side effects, such as resistance, secondary cancers, or toxicity to other healthy tissues [3]. The outcomes of common chemotherapies have been less than ideal, with the five‐year survival rate of such patients being approximately only 10% [4]. With the rapid development of immunotherapy strategies, therapeutics have evolved from cytokine‐based therapies to antibody blockade of cytotoxic T lymphocyte‐associated antigen‐4 (CTLA‐4) and programmed cell death protein 1 (PD‐1) immune checkpoints [5]. Although the treatment and survival rates for patients with melanoma have improved significantly, many patients still have no response or only a partial response to immunotherapy, and the majority of patients die from metastatic disease [6].

As a key component of the innate immune system, natural killer (NK) cells can quickly kill adjacent infected or cancerous cells [7] and enhance antibody‐mediated cytotoxicity [8] without antigen presentation. The activity of NK cells is primarily determined by the integration of signals from activating and inhibitory receptors [9]. Once activated, NK cells can kill targeted cells by secreting lytic granules (perforin and granzyme), cytokines with antitumor activity, and factors that recruit and promote the activation of other inflammatory cells that indirectly kill target cells [10]. Moreover, NK cells can also induce target cell apoptosis through Fas‐Fas ligand (FasL) and TRAI‐TRAI ligand (TRAIL) interactions [10]. However, the immunosuppressive properties of the tumor microenvironment (TME) indeed limit NK cell therapy [11, 12]. In addition, preclinical and clinical data have shown that the efficacy of NK cell therapy in solid tumors is worse than that in various leukemias, as several problems, including insufficient tumor infiltration and poor persistence/activation in the TME, exist [12, 13]. Growing clinical evidence suggests that an increased presence of NK cells is positively correlated with better survival rates in patients with multiple solid cancers and in the response to checkpoint blockade immunotherapy for patients with melanoma [14, 15].

The antitumor immunity of NK cells is regulated by a variety of factors, such as cytokines, ligand recognition, metabolism, and epigenetic modification [16]. A‐to‐I RNA editing is a posttranscriptional modification mechanism that can generate molecular diversity and not only has an important effect on the regulation of gene expression but is also closely related to the onset and development of many diseases [17]. ADAR1 catalyses the deamination of adenine (A) to inosine (I) in double‐stranded RNA (dsRNA) substrates that can reside in either coding or non‐coding regions, including 5′‐untranslated region (UTR), 3′‐UTRs, and introns. Recently, ADAR1 has been widely explored in several cancer immunotherapies, with results indicating that it may function as an immune checkpoint [18, 19]. Previous reports suggest that ADAR1 edits endogenous dsRNA for proper control of innate immune function [20]. ADAR1 mutations cause the development of a series of autoimmune diseases, which are generally accompanied by hyperactivated IFN‐stimulated genes (ISGs) and increased proinflammatory cytokine production [21]. Research on ADAR1 mutant mice has shown that ADAR1 is crucial for various physiological functions, including embryonic development [22], the immune response [23], B [23, 24] and T lymphocyte development [25], and CD103^+^ dendritic cell and alveolar macrophage development and function [26]. However, even though RNA editing is required for innate immune tolerance, the role of ADAR1 in NK cells is currently unknown.

In this study, we found that the ADAR1 expression level was increased in the PB‐NK cells of patients with melanoma and was positively correlated with the functional exhaustion of NK cells. ADAR1‐knockdown NK cells displayed increased antitumor activity in vitro and in vivo, accompanied by increased expansion and increased tumor tissue infiltration. Mice with conditional Adar1‐deficient NK cells presented impaired tumor progression and increased infiltration rates of NK cells into tumor tissue. RNA‐seq analysis revealed that ADAR1‐knockdown NK cells exhibited an activated phenotype with decreased inhibitory receptor expression and increased activation receptor expression, as well as increased migration‐related gene expression. Moreover, knocking down ADAR1 in NK cells resulted in increased cell mobility. In addition, we observed that ADAR1 deficiency in NK cells was accompanied by CD38 expression decline. Our study highlights ADAR1 as an emerging therapeutic target for enhanced NK cell immunotherapy.

## 2 Results

### 2.1 The Expression Level of ADAR1 was Elevated in the PB‐NK Cells of Patients with Melanoma

Even though NK cells possess strong tumor‐killing activity, the complexity of the TME restricts the efficiency of tumor therapy. Insufficient NK cell infiltration into tumors and the functional exhaustion of infiltrating NK cells play pivotal roles in this process. Microarray data of tumor‐infiltrated and tumor‐edge NK cells from patients with hepatocellular carcinoma (HCC) or NK cells from normal liver tissue revealed that the expression levels of effector function‐related genes and genes related to cell migration were decreased, whereas those of genes related to functional exhaustion were increased (Figure S1A). Previous genome‐wide analysis showed a negative correlation between RNA N6‐methyladenosine (m^6^A) and A‐to‐I RNA editing [27]. Song et al. reported that METTL3‐mediated m^6^A modification is essential for maintaining antitumor immunity of NK cells. Loss of Mettl3 in NK cells inhibited the infiltration and function of NK cells in the tumor microenvironment (TME), resulting in accelerated tumor development and shortened survival time of tumor‐bearing mice [28]. This indicated that, in contrast to the effect of m^6^A modification on NK cells’ function, upregulation of A‐to‐I editing could prejudice NK cells anti‐tumor activity. In mammalian cells, only ADAR1 and ADAR2 enzymes have the activity to bind and catalyze the A‐to‐I editing of double‐stranded RNA (dsRNA) [29, 30]. Notably, we observed that the *ADAR1* mRNA level was elevated by approximately 21.3% in the tumor‐infiltrating NK cells of HCC patients (Figure S1A), instead of *ADAR2* mRNA level. In addition, via the use of Gene Expression Profiling Interactive Analysis (GEPIA) online tools, we found that *ADAR1* mRNA expression was positively correlated with the module of the inhibitory receptor or suicide‐related genes of NK cells in different human tumors on the basis of the Cancer Genome Atlas (TCGA) and Genotype Tissue Expression (GTEx) databases (Figure S1B). Here, we aimed to explore the possibility of A‐to‐I editing mediated by ADAR1 as an immune checkpoint for NK cells.

**FIGURE 1:**
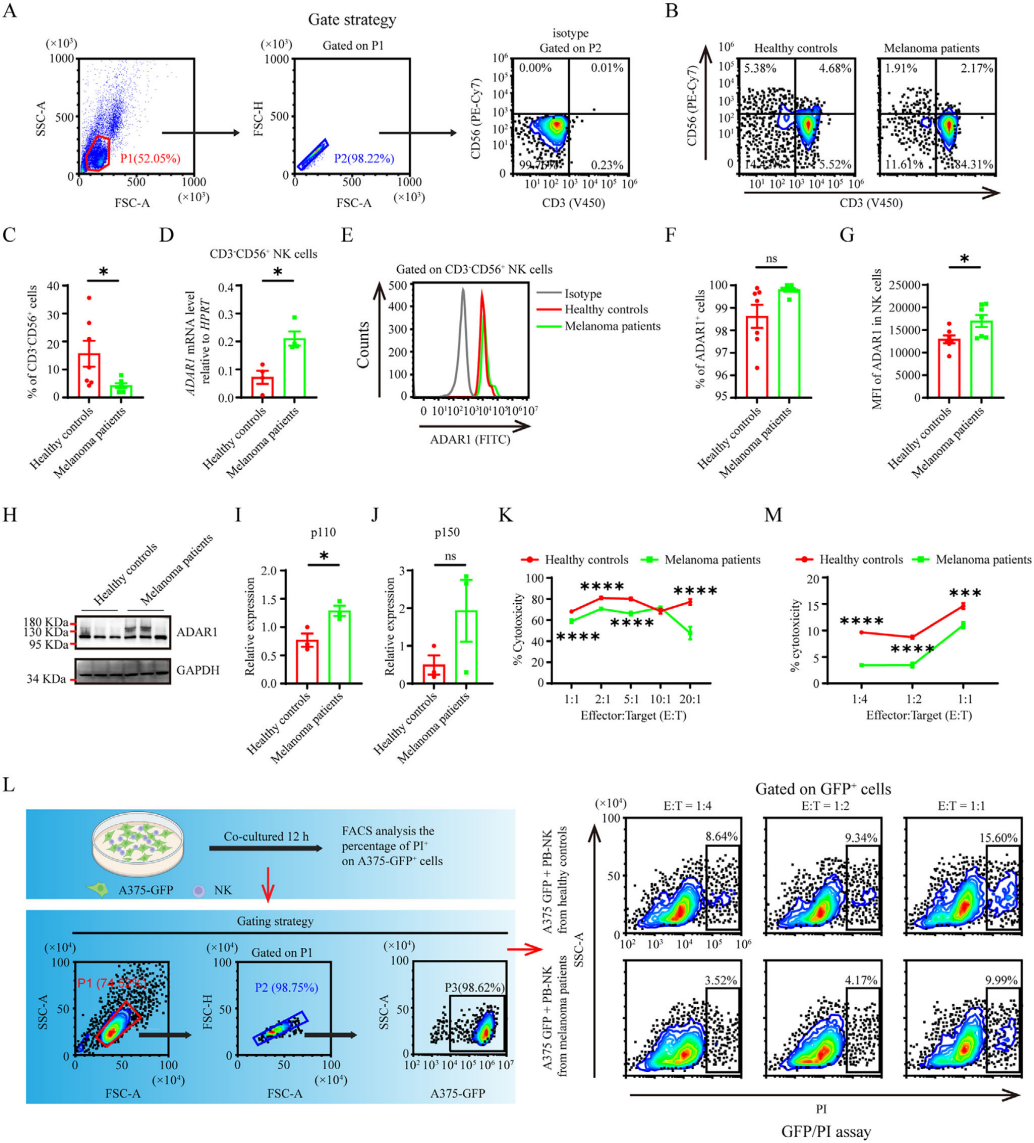
The expression level of ADAR1 was elevated in PB‐NK cells of patients with melanoma. Gating strategy (A), flow cytometry analysis (B) and statistical graph (C) showing the frequency of PB‐NK cells derived from healthy controls and melanoma patients (n = 7 for each group). mRNA level of ADAR1 in PB‐NK cells derived from healthy controls and melanoma patients (n = 4 for each group) (D). Flow cytometry analysis (E), statistical graphs of the percentage of ADAR1^+^ NK cells (F), and MFI (G) of ADAR1 in PB‐NK cells derived from healthy controls and melanoma patients (n = 7 for each group). Western blot analysis (H) and quantification of ADAR1 p110 (I) and p150 isoform (J) expression in PB‐NK cells derived from healthy controls and melanoma patients. LDH assay analysis of PB‐NK cells derived from healthy controls and melanoma patients toward A375 cells at different E:T ratios (n = 6 for each group) (K). Gating strategy of FACS analysis (L) and statistical graph (M) showing the cytotoxicity activity of PB‐NK cells derived from healthy controls and melanoma patients toward A375‐GFP cells at different E:T ratios (n = 3 for each group).

Since melanoma serves as a good example of how basic and translational medicine research can improve the prognosis of patients with cancer, some scientists believe that melanoma models can play an important role in the field of new therapeutic target discovery and innovative treatment strategies formulation [31, 32, 33, 34]. To confirm the upregulation of ADAR1 expression in NK cells during tumorigenesis, we first measured the frequency of PB‐NK cells derived from melanoma patients, and documented a significant decrease of CD3^−^CD56^+^NK cells relative to healthy controls, which was consistent with previous findings in patients with metastatic melanoma (Figure 1A–C) [35]. We then measured the mRNA and protein expression of ADAR1 in the PB‐NK cells of patients with melanoma. CD3^−^CD56^+^ PB‐NK cells were enriched via fluorescence‐activated cell sorting (FACS). The quantitative real‐time polymerase chain reaction (qRT‐PCR) data revealed an approximately 3‐fold increase in *ADAR1* mRNA expression in the PB‐NK cells of patients with melanoma compared with those of healthy donors (Figure 1D). Next, we detected ADAR1 protein expression via flow cytometry and western blotting. The percentage of ADAR1^+^ NK cells of patients with melanoma was slightly increased as compared with those of healthy controls (Figure 1E,F). The mean fluorescence intensity (MFI) of ADAR1 in PB‐NK cells derived from melanoma patients was also significantly greater than that in PB‐NK cells derived from healthy donors (Figure 1G). The western blot results exhibited that the expression level of the p110 isoform of ADAR1 was greatly increased in PB‐NK cells derived from melanoma patients as compared with those from healthy controls, instead of its p150 isoform (Figure 1H–J). Furthermore, we assessed their killing capacity of A375 cells, a melanoma cell line, via two different methods. Compared with PB‐NK cells derived from healthy controls, PB‐NK cells derived from melanoma patients exhibited impaired killing activity against A375 cells in a lactate dehydrogenase (LDH) cytotoxicity assay at different effector: target ratios (Figure 1K). A similar phenomenon was observed in the GFP/PI assay (Figure 1L,M). These results suggest that ADAR1 upregulation attenuated NK cell tumor control. The genetic deletion or pharmacologic inhibition of ADAR1 might prevent NK cell dysfunction, resulting in increased tumor killing.

### 2.2 ADAR1‐Knockdown NK‐92 Cells Displayed Enhanced Antitumor Activity In Vitro

**FIGURE 2:**
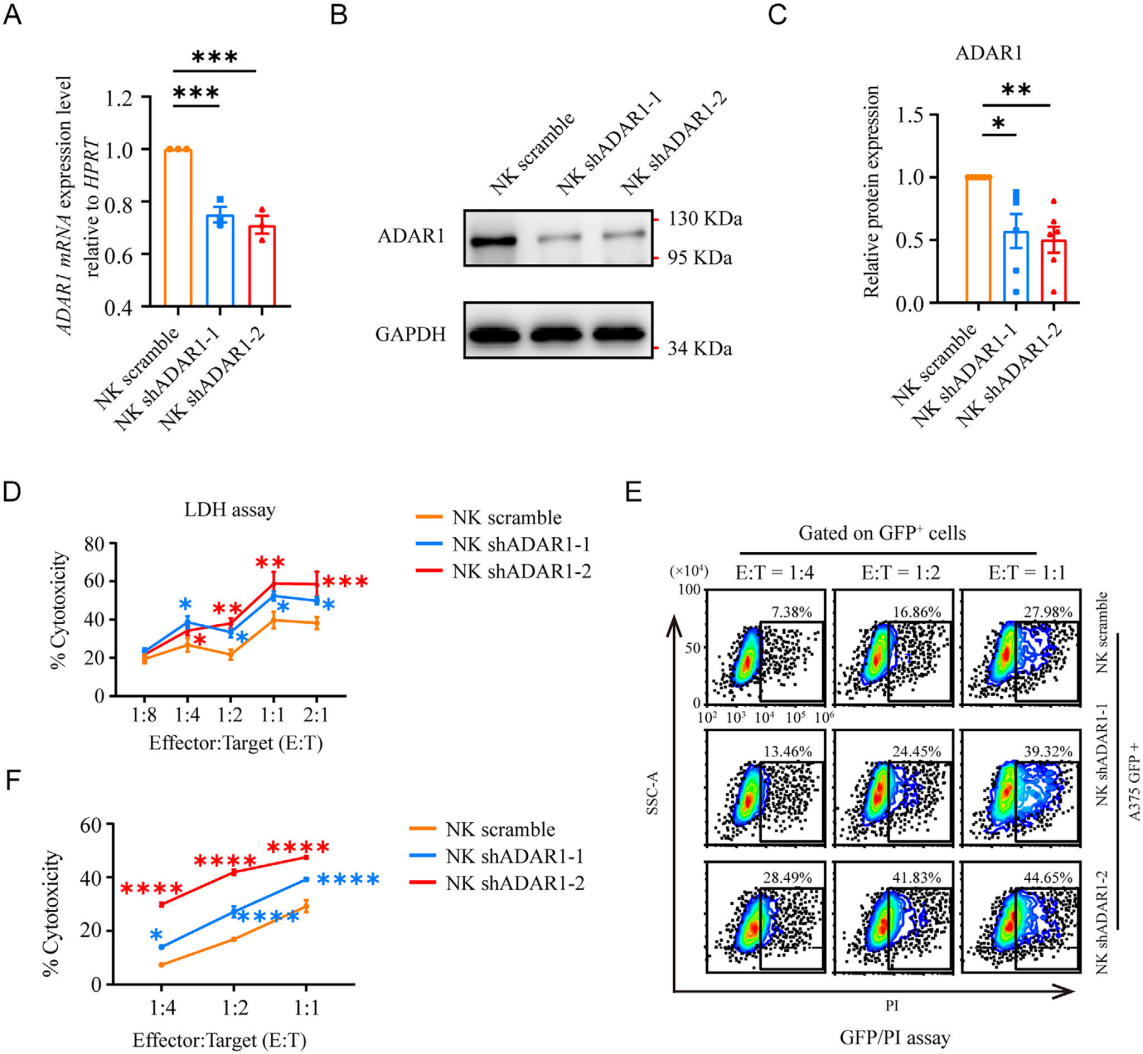
*ADAR1* knockdown promoted NK‐92 cells antitumor activity in vitro. qRT‐PCR analysis of NK cells after *ADAR1* knockdown (n = 3 for each group) (A). Western blot analysis (B) and quantification (C) of ADAR1 expression in indicated groups (n = 6 for each group). LDH assay analysis of NK scramble and NK shADAR1 cells toward A375 cells at different E:T ratios (n = 3 for each group) (D). FACS analysis (E) and statistical graph (F) showing the cytotoxicity activity of NK scramble and NK shADAR1 cells toward A375‐GFP cells at different E:T ratios (n = 3 for each group).

To verify whether ADAR1 knockdown promoted NK cell tumor control, we performed a cell cytotoxicity assay after ADAR1 expression was knocked down in NK‐92 cells, a widely used NK cell line. A vector containing scrambled shRNA was used as a negative control (NK scramble). First, the efficacy of ADAR1 knockdown in NK cells (NK shADAR1 cells) was confirmed via qRT‐PCR (Figure 2A) and western blotting (Figure 2B,C). Second, we compared their killing capacity of A375 cells. Compared with scramble cells, NK shADAR1 cells exhibited greater killing activity against A375 cells in a LDH cytotoxicity assay (Figure 2D) and GFP/PI assay (Figure 2E,F). Taken together, these results demonstrate that the downregulation of ADAR1 expression in NK cells leads to increased tumor‐killing activity.

### 2.3 ADAR1‐Knockdown NK‐92 Cells Displayed Increased Antitumor Activity In Vivo

**FIGURE 3:**
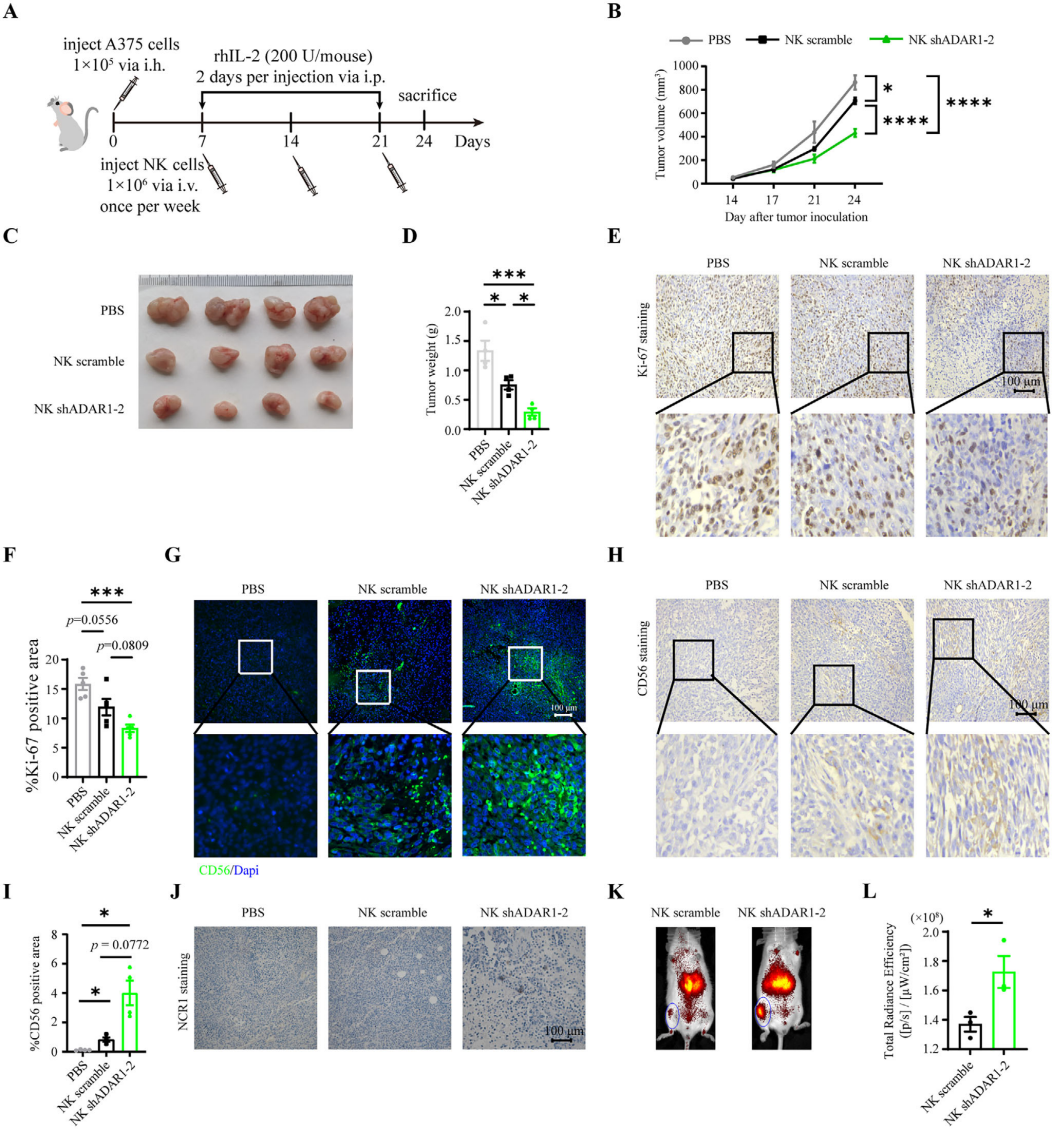
ADAR1 knockdown promoted NK‐92 cells infiltration and antitumor activity in vivo. Diagram of in vivo treatment scheme. NCG mice were hypodermic injected (i.h.) with 1 × 10^5^ A375 cells. 7 days later, mice were either untreated or treated with 1 × 10^6^ NK scramble or NK shADAR1 cells weekly. NK cells were supported injections of IL‐2 every two days for 3 weeks. At day 24, all mice were sacrificed then tumor tissues were dissected for further analysis (A). Tumor growth was monitored weekly by measuring tumor diameter using a vernier scale (n = 4 for each group) (B). Images of tumor tissues at day 24 (n = 4 for each group) (C). The weight of tumor tissues were measured at day 24 (n = 4 for each group) (D). Images of immunohistochemical staining of Ki‐67 in different groups tumor tissues. 5 random fields (200×) for each individual (E). Statistical graph showing the percentage of Ki‐67 positive area in different groups tumor tissues (F). Images of immunofluorescence staining of CD56 (green) and Dapi (blue) in different groups tumor tissues. 4 random fields (200×) for each individual (G). Images of immunohistochemical staining of CD56 in different groups tumor tissues. 5 random fields (200×) for each individual (H). Statistical graph showing the percentage of CD56 positive area in different groups tumor tissues (I). Images of immunohistochemical staining of human NCR1 in different groups tumor tissues (J). NCG mice were hypodermic injected (i.h.) with 1 × 10^5^ A375 cells. 21 days later, mice were treated with 1 × 10^6^ NK scramble or NK shADAR1 cells prestained with DIR dye. 24 h later, mice were anesthesitized with amiodarone, and then the distribution of NK cells in vivo was detected using Xenogen IVIS Spectrum Imaging Systems (K, n = 3 for each group). Total radiance efficiency of infiltrated NK cells at tumor location (L, n = 3 for each group).

To evaluate the antitumor activity of NK shADAR1 cells in vivo, mice were hypodermically injected (i.h.) with 1 × 10^5^ A375 tumor cells. Seven days later, the mice received a single intravenous (i.v.) injection of 1 × 10^6^ NK scramble or NK shADAR1‐2 cells per week (Figure 3A). As in previous studies [36], IL‐2 was administered every two days for 2 weeks to promote in vivo NK cell survival and expansion. Because the transplanted tumor tissue felt grainy for the first 2 weeks, we monitored tumor growth via a Vernier scale every other day on day 14 after tumor inoculation. Tumor growth was hampered in the NK scramble‐treated group and further inhibited in the NK shADAR1‐2‐treated group (Figure 3B). On day 24, the tumors in the phosphate buffer saline (PBS) group had grown to approximately 1000 mm^3^. All the mice were euthanized, and the tumors were collected and weighed. The mean weight of the tumors was lower in the NK scramble‐treated mice (0.753  ±  0.080 g) than in the PBS‐treated mice (1.334  ±  0.172 g). Among all the groups, the mean weight of the tumors in the NK shADAR1‐2 treatment group was the smallest (0.281  ±  0.054 g), which was corroborated by images of the tumors (Figure 3C,D).

To confirm therapeutic efficacy, dissected tumor tissues from different groups were analyzed by immunohistochemical staining for the antigen Ki‐67. As expected, positive staining, i.e., brown granules representing proliferative cells, was observed in the PBS group, but less positive staining was observed in the NK scramble cell‐treated group, especially in the NK shADAR1‐2 cell‐treated group (Figure 3E,F).

Another concern for NK cell adoptive immunotherapy is the infiltration rate or persistence of transplanted NK cells in tumor tissue, which is especially important for solid tumors. Tumor tissue was minced, and the infiltrated NK cells were analyzed via detecting the CD56 content in the different groups by immunofluorescence and immunohistochemical staining. There was no positive CD56 fluorescent signal in the PBS group, whereas there was a fluorescent signal in the NK scramble‐treated group, which was much more obvious in the NK shADAR1‐2‐treated group (Figure 3G). Consistent with these findings, immunohistochemical staining of CD56 revealed the same trend (Figure 3H,I). NCR1 immunohistochemical staining also exhibited similar results (Figure 3J). To further verify that ADAR1 knockdown was beneficial for NK cells’ tumor infiltration, NK scramble and NK shADAR1‐2 cells were stained with DIR dye before i.v. injection. The distribution of NK cells in vivo was then scanned with Xenogen IVlS Spectrum Imaging Systems. The result showed that the fluorescence intensity at the subcutaneous tumor site was higher in the NK shADAR1‐2 cell‐treated group as compared with NK scramble‐treated group (Figure 3K,L). Together, these results demonstrate that ADAR1‐knockdown NK‐92 cells have increased antitumor activity and persistence/infiltration in vivo.

### 2.4 Adar1 Deletion in NK Cells Enhanced the Control of Tumor Development

To validate that ADAR1 inhibited NK cell‐mediated antitumor immunity, we generated *ADAR1^flox/flox^*‐*Ncr1^iCre/+^* (*ADAR1* cKO) mice, which harbor a selective deficiency of ADAR1 in NK cells (Figure S2A,B). Prior to generating *ADAR1* cKO mice, we first confirmed the cutting efficacy of *Ncr1*‐*iCre*. We crossed GFP mice with *Ncr1‐iCre* mice. Without cutting, NK cells were GFP positive. After being cut, the NK cells were tdTomato positive. Six‐week‐old mice were sacrificed, and splenic CD3^−^NK1.1^−^ and CD3^−^NK1.1^+^ cells were used for analysis of the ratio of the tdTomato^+^ subpopulation. We found that approximately 85% of the splenic CD3^−^NK1.1^+^ cells were tdTomato positive, while there were no tdTomato positive signals observed in CD3^−^NK1.1^−^ cells, indicating effective cutting by *Ncr1‐iCre* (Figure S2C,D). The genotypes of newborn mice were monitored via genomic PCR of mouse tails (Figure S2E). To further confirm NK cell‐specific ADAR1 knockout, we sorted splenic CD3^−^NK1.1^+^ NK cells and CD3^−^NK1.1^−^ non‐NK cells from *ADAR1* WT and cKO mice and conducted genomic PCR (Figure S2F) and qRT‐PCR (Figure S2G,H) for *ADAR1*. The faint flox‐deletion signal observed in the CD3^−^NK1.1^−^ fraction aligns with the expected 1∼3% carry‐over of NK1.1^+^ cells during magnetic bead enrichment. The results revealed that the specific and efficient knockout of *ADAR1* occurred only in CD3^−^NK1.1^+^ cells.

**FIGURE 4:**
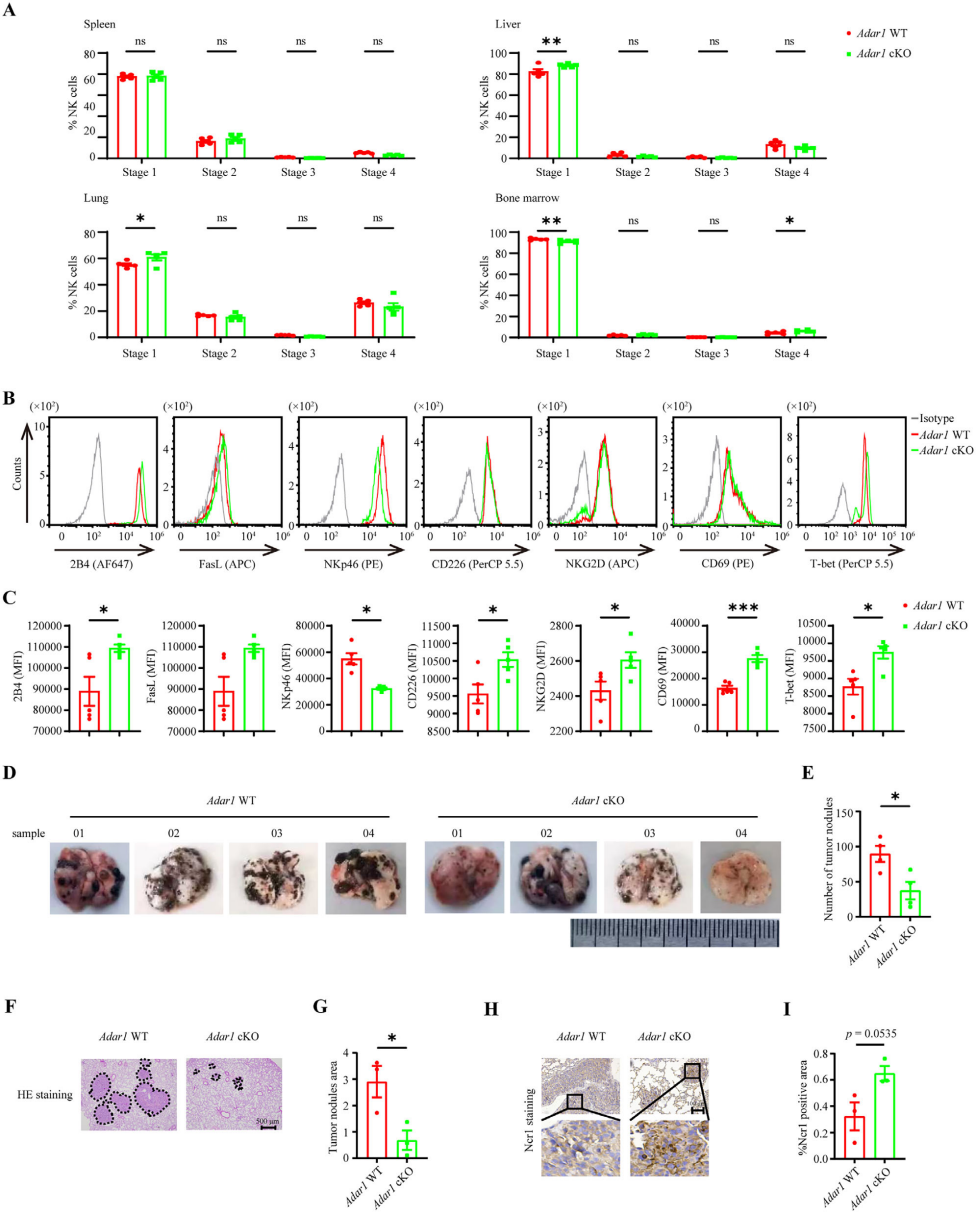
ADAR1 loss in NK cells gained better tumor development control. Percentages (A) of NK cells at different stages in the indicated organs of *Adar1* WT and cKO mice (n = 5 for each group). (Stage 1: CD27^−^CD11b^−^; Stage 2: CD27^+^CD11b^−^; Stage 3: CD27^+^CD11b^+^; Stage 4: CD27^−^CD11b^+^). Representative histograms (B) and MFI (C) of expression of the indicated molecules in splenic NK cells from *Adar1* WT and cKO mice (n = 5 for each group). Images of lungs (D) and statistical graph (E) of numbers of tumor nodules in lungs from *Adar1* WT and cKO mice 21 days after intravenous injection of 1 × 10^5^ B16 cells (n = 4 for per group). Representative HE staining images (F) and statistical graph (G) showing the tumor nodules area in the lung samples from *Adar1* WT and cKO mice after intravenous injection of 1 × 10^5^ B16 cells. 3∼5 random fields (200×) for each individual (n = 4 for each group). Immunohistochemical staining (H) and statistical graph (I) showing the percentage of Ncr1 positive area in lung samples from *Adar1* WT and cKO mice 21 days after intravenous injection of 1 × 10^5^ B16 cells (n = 3 for each group).

Prior to detecting the tumor control ability of *ADAR1* cKO mice, we first explored the effect of ADAR1 deletion on NK cell development (Figure 4A). In mice, maturation of NK cells is a four‐stage developmental program: CD27^−^CD11b^−^ (Stage 1) → CD27^+^CD11b^−^ (Stage 2) → CD27^+^CD11b^+^ (Stage 3) → CD27^−^CD11b^+^ (Stage 4). Compared with *ADAR1* WT mice, there was a significantly increased ratio of Stage 1 NK subsets in the spleen, liver, and lung in *ADAR1* cKO mice. However, the proportion of Stage 1 NK subsets in bone marrow was decreased, whereas the population of Stage 4 NK subsets was increased (Figure 4A). Moreover, the expression of the activating molecules 2B4, CD226, NKG2D, and CD69 was simultaneously induced in splenic NK cells from *ADAR1* cKO mice, while the expression of NKp46 was decreased (Figure 4B,C). In addition, ADAR1 deletion augmented the expression of the transcription factor T‐bet in splenic NK cells (Figure 4B,C). However, the level of apoptosis‐inducing molecule FasL in splenic NK cells derived from *ADAR1* WT and cKO mice was comparable (Figure 4B,C). These data reveal that even though ADAR1 deletion has different effects on the number and terminal maturation of NK cells in different organs, its deficiency promotes the expression of molecules related to effector functions.

Next, we sought to explore whether ADAR1 deletion influences the antitumor responses of NK cells in vivo. Compared with WT mice, *ADAR1* cKO mice intravenously injected with B16 cells presented significantly fewer lung tumor nodules, indicating less progressive pulmonary metastases (Figure 4D,E). Hematoxylin and eosin (HE) staining of lung tissue also revealed fewer tumor nodules in *ADAR1* cKO mice than in control mice (Figure 4F,G). Immunohistochemical staining of Ncr1 revealed that more NK cells infiltrated into tumor nodules in *ADAR1* cKO mice than in WT mice (Figure 4H,I). These results demonstrated that ADAR1 deficiency in NK cells resulted in better control of tumor growth.

### 2.5 ADAR1‐Knockdown NK‐92 Cells Displayed an Impaired Cytokine Storm Capacity to Favor its Proliferation

**FIGURE 5:**
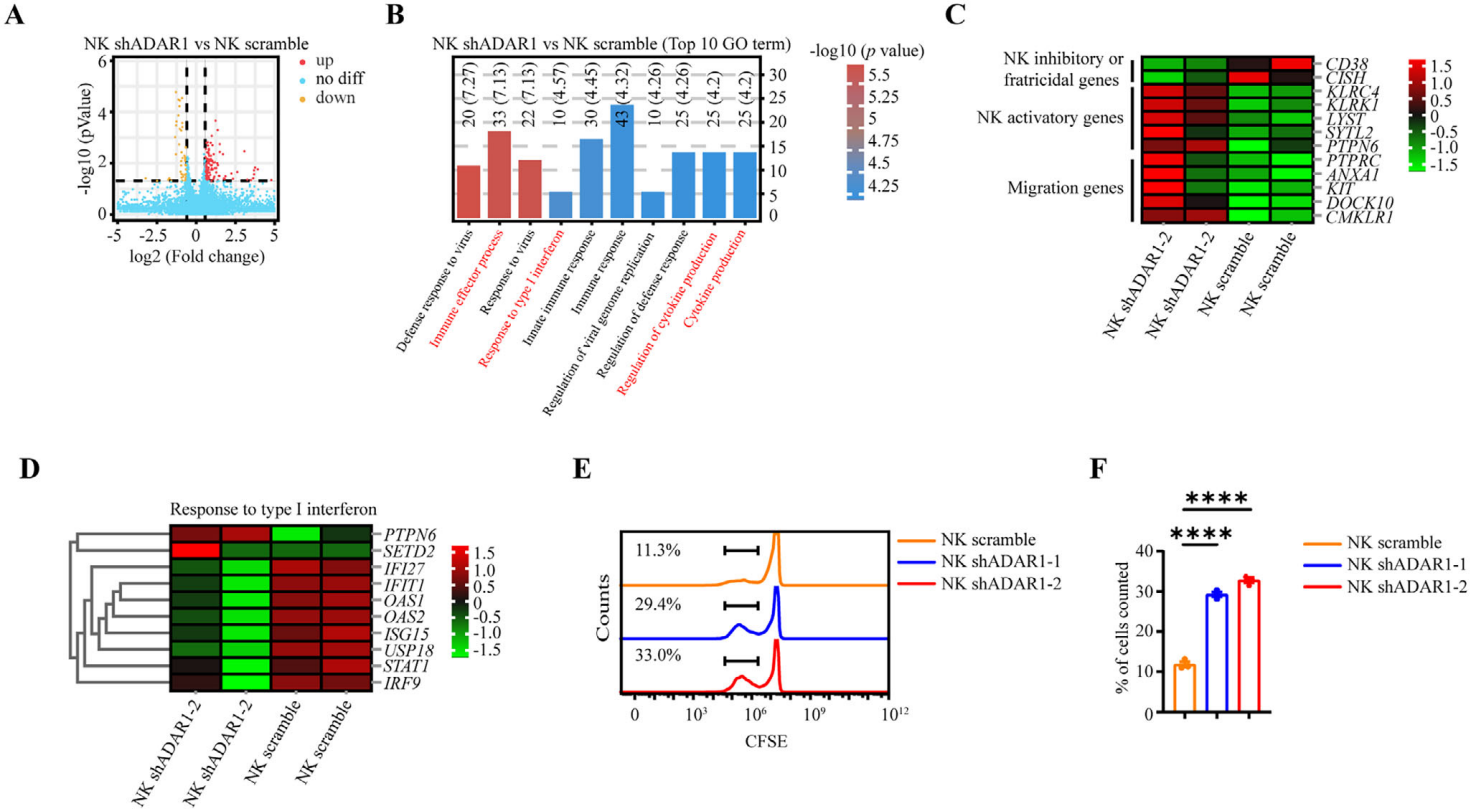
ADAR1 knockdown NK‐92 cells showed impaired cytokine storm to favor its proliferation. Volcano diagram of differential expression genes. The threshold of differential expression genes is: absolute fold change ≥ 1.5, *p*adj < 0.05 (A, n = 2 for each group). GO enrichment analysis. Top 10 significantly enriched biological process was shown (B, n = 2 for each group). Heatmap view of expression of 12 genes involved in NK inhibitory‐related, fratricidal‐related, activatory‐related and migration‐related in NK scramble and NK shADAR1 cells (C, n = 2 for each group). Heatmap view of expression of 10 genes involved in response to type I interferon in NK scramble and NK shADAR1 cells (D, n = 2 for each group). CFSE histograms (E) and statistical graph (F) of the indicated NK cells (n = 3 for each group).

Next, to investigate the molecular mechanisms leading to the increased tumor‐killing activity of ADAR1‐knockdown NK‐92 cells, we performed RNA sequencing on NK scramble and NK shADAR1‐2 cells. A total of 196 differentially expressed genes (DEGs, the threshold of differentially expressed genes was an absolute fold change ≥ 1.5 and *p*adj < 0.05) were identified in NK shADAR1‐2 cells (Figure 5A). Gene Ontology (GO) enrichment analysis of these DEGs suggested that the top 10 affected signaling pathways in NK shADAR1‐2 cells were associated with immune effector processes, response to type I interferon, regulation of cytokine production, and cytokine production (Figure 5B). NK shADAR1‐2 cells exhibited an activated phenotype, as shown by the decreased expression of the NK inhibitory receptor (*CISH*) and fratricidal‐related gene (*CD38*), increased expression of genes associated with NK cell effector function (*KLRC4*, *KLRK1*, *LYST*, *SYTL2*, and *PTPN6*), and increased expression of migration‐related genes (*PTPRC*, *ANXA1*, *KIT*, *DOCK10*, and *CMKLR1*) (Figure 5C).

Previous studies reported that the deletion or inactivation of ADAR1 initiates a cytokine storm, endoplasmic reticulum (ER) stress, and apoptosis in different cell types [37, 38, 39]. Surprisingly, according to our RNA‐seq data, the GO term analysis revealed the downregulation of type I interferon target genes (Figure 5D), with no significant changes in NF‐κB signaling or apoptosis (Figure S3A,B). So, we evaluated the proliferative potential and apoptotic effect of NK shADAR1 cells. We found that NK shADAR1 cells possessed an increased proliferation capacity without significant effects on apoptosis (Figure 5E,F; Figure S3C,D). Interestingly, Gene Set Enrichment Analysis (GSEA) revealed that genes involved in the RIG‐I‐like receptor signaling pathway, as well as those involved in cytosolic DNA sensing, were significantly repressed in NK shADAR1‐2 cells, and no change was observed in JAK‐STAT signaling(Figure S3E–G). The western blot results showed that there was lower RIG‐I and MDA5 expression in NK shADAR1 cells than in NK scramble cells. However, the expression of RIG‐I was rescued when overexpression of ADAR1 in NK shADAR1 cells, but not MDA5 (Figure S3H–K). The ADAR1 inhibitor 8‐Aza suppresses RIG‐I and MDA5 RNA levels in NK‐92 cells (Figure S3L,M). The suppression of RIG‐I was also observed in NK cells derived from *ADAR1* cKO mice (Figure S3O,P). Collectively, we speculated that the downregulation of ADAR1 expression in NK‐92 cells does not lead to an acute cytokine storm to favor its proliferation.

### 2.6 ADAR1 Deficiency in NK‐92 Cells Promoted their Mobility Toward Tumors

**FIGURE 6:**
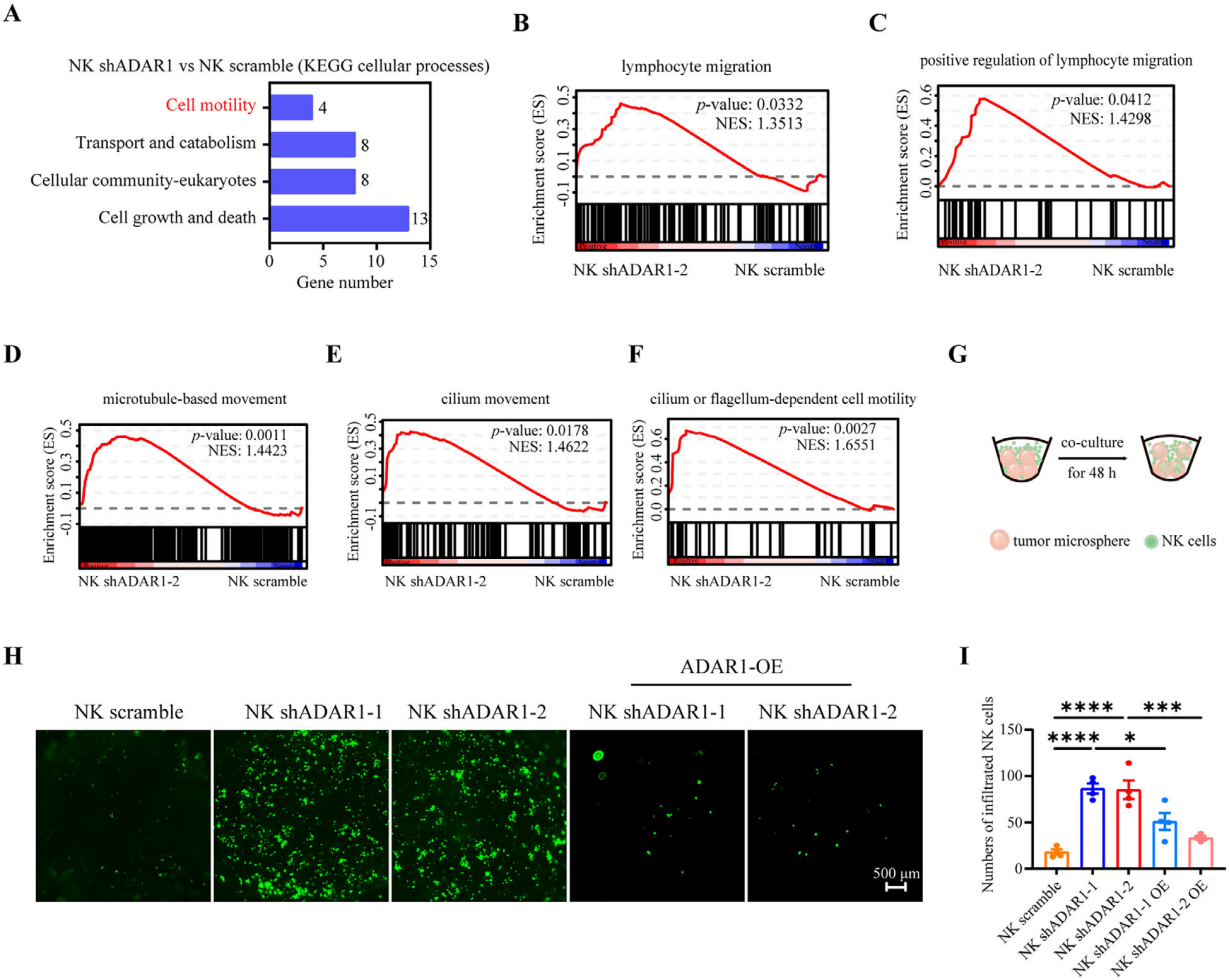
ADAR1 deficiency in NK‐92 cells promoted its mobility toward tumor location. KEGG analysis. 4 cellular processes affected by ADAR1 knockdown were showed (A). GSEA result of lymphocyte migration (B), positive regulation of lymphocyte migration (C), microtubule‐based movement (D), cilium movement (E), and cilium or flagellum‐dependent cell motility (F). Schematic diagram of evaluating NK cells tumor infiltration activity. A375 cells were embedded in 2×Matrigel, further cultured for 48 h and incubated with CFSE stained NK scramble or NK shADAR1 cells for another 48 h. After removing the un‐infiltrated cells, photos were taken (G). Z‐stack images (maximum intensity projection) showing infiltration of NK scramble and NK shADAR1 cells into 3D tumor microsphere (4×, n = 6 for NK scramble and shADAR1 groups, n = 8 for ADAR1 overexpression groups) (H). Statistical graphs showing the numbers of infiltrated NK cells (n = 6 for NK scramble and shADAR1 groups, n = 8 for ADAR1 overexpression groups) (I).

Kyoto Encyclopedia of Genes and Genomes (KEGG) analysis of the RNA‐seq data revealed that cell motility was one of 4 cellular processes affected by ADAR1 knockdown (Figure 6A). GSEA also revealed that cell motility‐related genes, such as those related to lymphocyte migration, the positive regulation of lymphocyte migration, microtubule‐based movement, cilium movement, and cilium‐ or flagellum‐dependent cell motility, were enriched in NK shADAR1 cells (Figure 6B–F). To verify this observation, a 3D protocol was adapted to form tumor microspheres, which were further cocultured with carboxyfluorescein succinimidyl ester (CFSE)‐stained NK‐92 cells. After removing noninfiltrated NK cells, photographs of the tumor microspheres were taken (Figure 6G). The results revealed that knocking down ADAR1 increased NK‐92 cell infiltration into tumor microspheres (Figure 6H,I), which was consistent with the observations in ADAR1‐knockdown NK‐92 cells (Figure 3G–L) and *ADAR1* cKO mice (Figure 4H,I). Moreover, overexpression of ADAR1 in NK shADAR1 cells decreased the infiltration rate (Figure 6H,I). These results suggested that a lack of ADAR1 in NK‐92 cells improved their ability to favor tumor tissue infiltration to gain better control of tumor development.

### 2.7 NK Cells Lacking ADAR1 Expression were Accompanied by a Decrease in CD38 Expression

**FIGURE 7:**
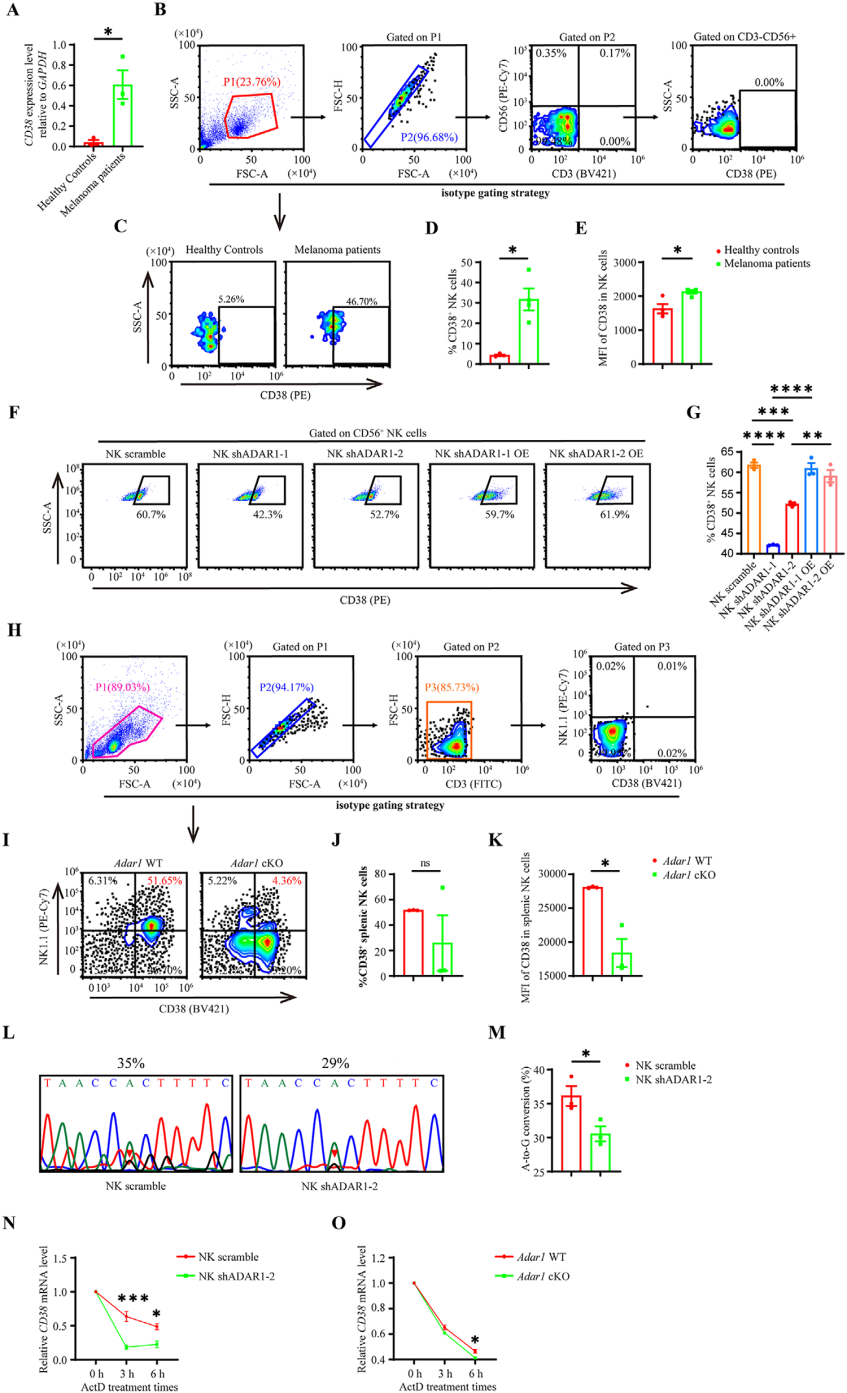
NK cells lacking of ADAR1 accompanied by CD38 expression decline. mRNA level of *CD38* in PB‐NK cells of healthy donors and melanoma patients (n = 3 for each group) (A). Flow cytometry analysis strategy of CD38 expression (B). Flow cytometry analysis (C), statistical graph showing the percentage (D), and MFI of CD38 (E) in PB‐NK cells derived from healthy donors and melanoma patients (n = 4 for each group). Flow cytometry analysis (F), statistical graph showing the percentage (G) of CD38 in indicated cells (n = 3 for each group). Flow cytometry analysis strategyof CD38^+^ NK cells (H). Flow cytometry analysis (I), statistical graphs of the percentage (J), and MFI of CD38 (K) in splenic NK cells from *Adar1* WT and cKO mice (n = 3 for each group). Sequence chromatograms (L) and statistical graph (M) of the CD38 transcript in the indicated cell groups (n = 3 for each group). The relative *CD38* mRNA level in NK scramble and shADAR1‐2 cells treated with or without ActD (5 µg/ml) (N, n = 3 for each group). The relative *CD38* mRNA level in splenic NK cells derived from *Adar1* WT and cKO mice treated with or without ActD (5 µg/ml) (O, n = 3 for each group).

CD38 is a transmembrane glycoprotein widely expressed by many cells. It was reported to be tightly coexpressed with the exhaustion marker PD‐1 [40]. As a nicotinamide adenine dinucleotide (NAD^+^) consumer, a relatively high level of CD38 in CD8^+^ T cells is associated with NAD^+^ depletion, resulting in CD8^+^ T cell exhaustion [41, 42]. NAD^+^ precursor nicotinamide (NAM) supplementation enhances the tumor‐eliminating function of T cells both in vitro and in vivo [43]. An inverse correlation between CD38 and NAD^+^ determines long‐term T cell‐mediated cytotoxicity [40]. Given the similar genome‐wide epigenetic and transcriptional profiles between adaptive NK cells and CD8^+^ T cells [44, 45], and prompted by our RNA‐seq data implicating CD38 in NK cell function, we first compared the CD38 expression levels in PB‐NK cells derived from healthy donors and patients with melanoma. The qRT‐PCR results revealed that the mRNA level of *CD38* was increased in the PB‐NK cells of patients with melanoma (Figure 7A). The frequency of CD38^+^ NK cells and the MFI of CD38^+^ NK cells also exhibited similar trends, which were similar to previous findings that CD38 expression was upregulated in tumor‐infiltrated CD8^+^ T cells (Figure 7B–E) [42]. Second, we measured the CD38 expression level after ADAR1 knockdown. The frequency of CD38^+^ NK‐92 cells was significantly decreased when knocking down the expression of ADAR1 in NK‐92 cells, but significantly increased when overexpression of ADAR1 in NK shADAR1 cells (Figure 7F,G). Finally, we checked the Cd38 expression level in splenic NK cells derived from *ADAR1* WT and *ADAR1* cKO mice. The results revealed that although the percentage of CD38^+^ splenic NK cells was comparable between *ADAR1* WT and cKO mice, the MFI of CD38 in splenic NK cells derived from *ADAR1* cKO mice was significantly lower than that in those derived from *ADAR1* WT mice (Figure 7H–K).

To test whether CD38 acted as a potential substrate of ADAR1, we analyzed the single nucleotide polymorphism (SNPs) in our RNA‐seq data. Total 6 SNPs sites at 3’‐UTR region of CD38 were identified in our RNA‐seq data. We selected one of these mutations, A>G (15852061), for validation. This modification was confirmed by Sanger sequencing (Figure 7L) [46]. The abundance of this modification from the Sanger sequence was also quantified using the BEAT program (https://hanlab.cc/beat/). The result showed that the frequency of guanine at this site was reduced in NK shADAR1‐2 cells, as compared with that in NK scramble cells (Figure 7M). mRNA stability assay indicated that the stability of CD38 mRNA in NK shADAR1‐2 cells was lower than that in NK scramble cells (Figure 7N). A similar result was observed in splenic NK cells derived from *ADAR1* WT and cKO mice (Figure 7O). These results indicate that CD38 may act as a potential substrate of ADAR1. The deficiency of ADAR1 in NK cells is accompanied by a downregulation of CD38 expression.

## 3 Discussion

The present study was designed to determine the role of ADAR1 in enhancing the NK‐mediated killing of melanoma cells. On the basis of previous RNA‐seq data, we found that the expression of ADAR1 was upregulated in tumor‐infiltrated NK cells. Next, we discovered the excellent antitumor activity and cell mobility potential of ADAR1‐deficient NK cells in vitro and in vivo. Mechanistically, ADAR1 deficiency in NK cells is accompanied by CD38 expression decline via affecting its mRNA stability, resulting in increased cell mobility, proliferation, and tumor killing capacity.

In the last decade, considerable efforts related to immune checkpoints have been made to improve the antitumor activity of NK cells. However, the ability of NK cells to kill solid tumors is still not satisfactory. We analyzed previous data from the Gene Expression Omnibus (GEO) dataset (GSE120123) and found that NK cells in tumor tissues presented increased *ADAR1* expression. Next, we confirmed that the ADAR1 expression level in NK cells from patients with melanoma was indeed higher than that in NK cells from healthy donors by testing peripheral blood samples. We found that knocking down ADAR1 expression enhanced the tumor‐killing function of NK cells in vitro and in vivo. Improved control of tumor development was also discovered in *ADAR1* cKO mice. Our RNA‐seq data indicated that when ADAR1 was knocked down in NK‐92 cells, type I IFN signal activation was not detected. In contrast, the expression of genes related to this pathway was downregulated. This finding is not consistent with previous research [21]. Hachung et al. speculated that the reason for this distinction was that ADAR1‐mediated negative type I IFN or ISG expression occurred in a cell type‐specific manner [47]. In addition, the absence of *ADAR1* in mouse liver cells and endothelial cells can lead to NF‐κB signal activation and cell apoptosis [48, 49]. However, our data show that ADAR1 knockdown does not increase NK cell apoptosis. Next, we observed that ADAR1 knockdown in NK cells led to decreased RIG‐I expression. Previous studies have reported that ADAR1 restricts RIG‐I/MDA5‐mediated dsRNA immune sensing [50, 51, 52]. Notably, work from the Rice group indicated that ADAR1 dsRNA binding and catalytic activities were required to block translational shutdown [47], knocking out ADAR1 or inhibiting its enzymatic activity may both cause translation shutdown. Thus, loss of ADAR1 may broadly suppress protein synthesis, offering a plausible explanation for the concomitant downregulation of RIG‐I. This interpretation aligns with independent findings from the Lu group, which identified RIG‐I as a negative regulator of CD8^+^ T‐cell anti‐tumor function and showed that adoptive transfer of RIG‐ deficient CD8^+^ T cells enhances tumor clearance [53], consistent with our observation that diminished RIG‐I correlates with heightened NK cell activity. Although the precise mechanism by which ADAR1 regulates RIG1 in NK cells has yet to be determined, our collective findings suggest that the functional outcome of ADAR1 manipulation may represent a cell type‐specific phenomenon.

Furthermore, we found that the expression level of CD38 in NK cells was reduced in NK cells lacking ADAR1 expression. CD38 plays a role in the generation of an immunosuppressive TME. Extracellular NAD^+^ can generate AMP under the action of CD38 and CD203a, which are then hydrolyzed by CD73 to produce adenosine (ADO). ADO participates in immune suppression by binding to purinergic receptors [54]. Chatterjee et al. reported that a decrease in CD38 surface expression and an increase in NAD^+^ levels on T cells can enhance the antitumor ability of adoptive T cells, greatly improving tumor control. The strategy of targeting the CD38‐NAD^+^ axis can improve the efficacy of antitumor adoptive T‐cell therapy [40]. CD38 deletion in NK cells eliminates Dara‐mediated fratricide and enhances the antitumor activity of NK cells [55]. These findings suggest that CD38 may serve an immune checkpoint function [56]. Moreover, the temporary upregulation of CD38 was observed in CD8^+^ cytotoxic T cells treated with checkpoint inhibitors (such as PD‐1/PD‐L1 blocking antibodies) [41]. The increase in CD38 activity, in turn, leads to the inhibition of CD8^+^ T cells [57]. Similarly, the phenotype in which CD56^bright^CD16^−^ NK cells produce ADO and inhibit autologous CD4^+^ T cell proliferation can be restored with CD38 inhibitors [58]. Moreover, Chatterjee et al. reported that Treg cells and MDSCs isolated from CD38 knockout mice exhibited decreased functional inhibition, whereas activated CD38 knockout NK cells secreted increased levels of effector cytokines [40]. The discovery that this decrease in CD38 expression is beneficial for cell proliferation is consistent with our observations that ADAR1 knockdown in NK cells is accompanied by a decrease in CD38 expression and improved cell proliferation ability.

In addition, given a recent report that impaired NAD^+^ salvage in tumor‐infiltrating NK cells is correlated with poor patient survival and that supplementation with the NAD^+^ precursor nicotinamide mononucleotide (NMN) significantly promotes the antitumor activity of both human and mouse NK cells [59], we hypothesized that the intracellular NAD^+^ level increases in ADAR1‐deficient NK cells due to CD38‐mediated NAD^+^ consumption blockade, which favors the antitumor efficacy of ADAR1‐deficient NK cells. Since CD38‐knockout NK cells are relatively resistant to oxidative stress and CD38‐mediated NK cell fratricide [60, 61], combination strategies involving ADAR1‐deficient NK cells and anti‐CD38‐targeting monoclonal antibodies could be used in tumor therapy.

## 4 Conclusion

In summary, we demonstrated that NK cells are essential for anti‑tumor activity against melanoma. During this process, ADAR1, a previously identified RNA‐editing enzyme, also represents a checkpoint regulating NK cell function. These results shed light on the importance of NK cells in tumor immunity and suggest that targeting ADAR1 might be a promising strategy to enhance NK cells‐based immunotherapy for improved tumor control.

## 5 Experimental Section

### 5.1 Patients and Healthy Donors

This study was approved by the Ethics Committee of the Third Affiliated Hospital of Sun Yat‐sen University (approval number: [2022]02‐329‐01). Peripheral blood mononuclear cells (PBMCs) were obtained from patients who provided informed consent. PBMCs were also obtained from healthy volunteers who were age‐ and sex‐matched to the patients. All the PBMCs were cryopreserved in serum‐free cell freezing medium (New Cell & Molecular Biotech, C40100) before analysis. Detailed patient information is provided in Table S1.

### 5.2 Mice

Six‐week‐old male NCG (T001475) mice lacking mature T, B, and NK cells were purchased from GemPharmatech Co., Ltd., Nanjing, China, as were GFP mice (CAG‐LoxP‐ZsGreen‐Stop‐LoxP‐tdTomato mice, T006163), *ADAR1^flox/flox^* mice (T008358), and *Ncr1*‐*iCre*‐P2A mice (T005674). *Ncr1* ^iCre/+^^;^ *ADAR1* ^flox/flox^ mice (*ADAR1* cKO) were generated by crossing *Ncr1‐iCre* mice with *ADAR1^flox/flox^* mice. *ADAR1^flox/flox^* littermates were used as WT controls. All the mice were maintained under specific pathogen‐free conditions and provided autoclaved food and water in the animal center at Sun Yat‐sen University. All animal experiments were approved by the Ethics Committee of Sun Yat‐sen University (approval numbers: SYSU‐IACUC‐2022‐000307 and SYSU‐IACUC‐2023‐001504). The primers used for genotyping are listed in Table S2.

### 5.3 Cell Lines and Plasmids

The 293T, A375, and B16 cell lines were cultured in DMEM (C11995500BT, Gibco) supplemented with 10% heat‐inactivated fetal bovine serum (FBS, ExCell Bio, FSP100) and 1% penicillin‐streptomycin solution (KGY0023, KeyGEN). The NK‐92 cell line was cultured in RPMI 1640 medium (11875119, Gibco) supplemented with 12.5% FBS, 50 µm β‐mercaptoethanol (21985023, Gibco), 100 IU/mL recombinant human IL‐2 (S20020004, Qiagen), and 1% penicillin‐streptomycin solution. All cells were confirmed to be free of mycoplasma contamination.

Scramble shRNA and ADAR1‐targeting shRNA‐containing pLKO.1 lentiviral plasmid was constructed according to the protocol provided by the Addgene website. The sequences of shRNA1 and shRNA2 for human ADAR1 are listed in Table S3. The full‐length ADAR1 was cloned into pCDH‐CMV vector to form ADAR1 overexpression (OE) plasmid.

### 5.4 Isolation and Expansion of PB‐NK Cells

Mononuclear cells were isolated through density gradient centrifugation, and NK cells were enriched using the human NK Cell Serum‐free Culture Kit (NC0102.F, NC0102, and AN0104, YOCON).

### 5.5 Isolation and Expansion of Splenic NK Cells

The splenic NK cells were enriched using EasySep Mouse NK Cell lsolation Kit (19855, Stemcell) following the manufacturer's instructions. Then, the isolated NK cells were expanded using the human NK Cell Serum‐free Culture Kit (NC0102.F, NC0102, and AN0104, YOCON).

### 5.6 Lentivirus Packaging and Infection

To generate lentiviruses to infect NK‐92 cells, a three‐plasmid system was used. pLKO.1‐puro expressing ADAR1‐specific shRNA or the corresponding control plasmid, psPAX2 packaging plasmid, and pMD2G envelope plasmid were transfected into 293T cells at a 3:2:1 ratio via polyethylenimine (PEI, HY‐K2014, MCE) transfection. Cell culture supernatants containing the lentiviruses were collected 48 h after transfection and filtered through a 0.45 µm nonpyrogenic filter (FPV403150, JET BlOFIL). For transduction, lentivirus was added to NK‐92 cells in the presence of 8 µg/mL polybrene (HY‐112735, MCE). The samples were subsequently centrifuged at 1800 g for 60 mins at 32°C and incubated overnight in a humidified incubator at 37°C with 5% CO_2_ before the medium was replaced with regular growth medium [28]. The efficiency of the knockdown was verified via qRT‐PCR, western blotting, and flow cytometry (CytoFLEX, Beckman Coulter) on the 4th or 5th day after lentivirus infection.

### 5.7 Cytotoxicity Functionality Assay—LDH Cytotoxicity Assay

As described in a previous study [62], the human melanoma cell line A375 was seeded as target cells in 96‐well plates (5 × 10^3^ cells per well) in a total volume of 100 µL of DMEM supplemented with 10% heat‐inactivated FBS per well and cultured in a humidified incubator at 37°Cwith a 5% CO_2_ atmosphere for 12 h. The cells were then divided into five different groups as follows: reagent correction (no cells), target cells with spontaneous LDH efflux, target cells with maximum LDH efflux, effector NK cells with spontaneous LDH efflux, and NK/cancer cell coculture. For the NK cells in the spontaneous LDH efflux and coculture groups, NK cells were resuspended in 100 µL of NK medium without FBS and then added to the corresponding wells. The same medium was added to the other wells. Twelve hours later, approximately 20 µL of lysis buffer (10×) was added to the wells containing the target cells with maximum LDH efflux, which were subsequently incubated in a 37°C incubator for 30 mins. After centrifugation at 250 g for 3 mins, the cell culture supernatant was used for the LDH cytotoxicity assay (CK12, DOJINDO) to measure cell death following the manufacturer's instructions and using a Biotek ELX 800 plate reader.

### 5.8 Cytotoxicity Functionality Assay—GFP/PI Assay

A cytotoxicity assay of NK cells against melanoma cells was carried out via flow cytometry. The melanoma cell line A375 was infected with the GFP virus and treated with puromycin (1 µg/mL, HY‐K1057, MCE) for 7 days to generate A375‐GFP stable cells, which were used as target cells. These cells were cocultured with effector NK‐92 cells or PB‐NK cells derived from healthy controls and patients with melanoma at various effector:target ratios. After 12 h of incubation, all the cells in the mixture were collected and stained with propidium iodide (PI, 556547, BD) for 15 mins at room temperature and immediately analyzed via flow cytometry. The background was obtained by incubating target cells with medium. The maximum PI staining was calculated as 100%. The percent specific cytotoxicity was calculated as follows: (% PI^+^GFP^+^ cells in coculture ‐ % PI^+^GFP^+^ cells in background)/(100% ‐ % PI^+^GFP^+^ cells in background) × 100.

### 5.9 Cell Proliferation and Apoptosis Assays

5, 6‐carboxyfluorescein diacetate succinimidyl ester (CFSE, C34554, Invitrogen, USA) was dissolved at 5 mm in DMSO and stored at −80°C. Cells were washed once with PBS, resuspended in PBS containing 5 µm CFSE, incubated for 10 mins at 37°C in the dark, washed 3 times with PBS, and resuspended in NK‐92 medium. A total of 2 × 10^5^ cells in 200 µL of NK‐92 medium were seeded into a flat‐bottom 96‐well plate. Four days later, the cells were collected and used for flow cytometry analysis. For the apoptosis assay, cells were washed once with cold PBS, resuspended in 100 µL of 1×binding buffer (supplemented with 5 µl of FITC anti‐Annexin V, 556547, BD), and then incubated at room temperature (RT) for 15 mins in the dark. Five minutes before analysis, 300 µL of 1×binding buffer and 5 µL of PI (556547, BD) were added to each tube. The samples were analyzed by flow cytometry within 1 h. The results were analyzed with CytExpert software (Beckman Coulter) and Flowjo software （BD Biosciences).

### 5.10 Flow Cytometry

Flow cytometry was performed on a Beckman Coulter instrument, and the data were analyzed using CytExpert software. The antibodies used in this study included a V450‐conjugated anti‐CD3 antibody (560365, BD), a FITC‐conjugated anti‐CD3 antibody (300306, Biolegend), a PE‐Cy7‐conjugated anti‐CD56 antibody (362510, Biolegend), an APC‐conjugated anti‐CD56 antibody (362504, Biolegend), a PE‐conjugated anti‐CD38 antibody (555460, BD), and a Brilliant Violet 421‐conjugated anti‐CD38 antibody (102732, Biolegend).

For flow cytometry analysis of ADAR1, cells were collected and incubated with fixation/permeabilization solution (554714, BD) at 4°C for 20 mins. After being washed with 1×Perm/Wash buffer, the cells were incubated with an anti‐ADAR1 antibody (5 µg/ml; sc‐73408; Santa Cruz Biotech) at 4°C for 30 mins, washed with PBS twice, incubated with Alexa Fluor 488‐conjugated goat anti‐mouse IgG (1:200, A‐11001, Thermo) at 4°C for 30 mins, and finally analyzed with a Beckman Coulter machine. The antibodies used are listed in Table S4.

### 5.11 Immunofluorescence

Tissue samples obtained from mice were fixed in 4% formalin, embedded in paraffin, and cut into 5 µm sections. For CD56 immunofluorescence staining, the sections were deparaffinized, rehydrated, and pretreated via heat‐mediated antigen retrieval with Tris/EDTA buffer (pH 9.0) for 20 mins. The sections were blocked in 3% BSA (supplemented with 0.2% Triton X‐100) and then incubated with CD56 (1:650, GB12041, Servicebio) overnight at 4°C, followed by incubation with Alexa Fluor 488‐conjugated goat anti‐rabbit IgG (1:200, A‐11008, Thermo). Fluorescence images were captured using a microscope (Nikon, Japan). The antibodies used are listed in Table S4.

### 5.12 Immunohistological Analysis

After antigen retrieval, the endogenous peroxidase activity in sections was inactivated with 3% H_2_O_2_ at RT for 15 mins and then incubated with anti‐CD56, anti‐Ki‐67 (1:1000, ab15580, Abcam), and anti‐NCR1 (1:100, ab283505, Abcam) antibodies as described above, followed by incubation with a secondary antibody. The color was developed by incubation with a Dako Real^TM^ kit (K5007, Dako), and the sections were scanned under a microscope (Nikon, Japan). The positive areas were quantified in 3∼5 random fields (200×) for each individual.

### 5.13 RNA Extraction and qRT‐PCR

Total RNA was extracted from cells using TRIzol reagent (15596026, Invitrogen) and reverse transcribed into cDNA using the HiScript III RT SuperMix for qPCR (+gDNA wiper) Kit (R323, Vazyme). The relative expression levels of mRNAs were determined using qRT‐PCR with a LightCycler 480 instrument (Roche) and ChamQ SYBR Color qPCR Master Mix (Q421, Vazyme). The results were analyzed via the *ΔΔ*Ct method. The sequences of the forward and reverse primers (2.5 µm) for human *HPRT*, *CD38*, *ADAR1*, and *GAPDH* are listed in Table S5, as well as primers for mouse *Hprt*, *ADAR1*, *CD38*, and *Gapdh*.

### 5.14 Western Blot Analysis

Cells were harvested, lysed, and separated via SDS‐PAGE. Immunoblotting was carried out with the following primary antibodies: anti‐ADAR1 (1:2000, sc‐73408; Santa Cruz Biotech. Inc.) and anti‐GAPDH (1:2000, 5174S, Cell Signaling Technology). The following secondary antibodies were used: goat anti‐mouse IgG (1:2000, 7076, Cell Signaling Technology) and goat anti‐rabbit IgG (1:2000, 7074, Cell Signaling Technology). All images were acquired using a ChemiDoc XRS system (Bio‐Rad, CA, USA).

### 5.15 RNA‐seq and Bioinformatics Analysis

Total RNA was isolated from cells using TRIzol reagent (15596026, Invitrogen) according to the manufacturer's protocol. RNA quality control, library construction, sequencing, and data analysis were performed using an Illumina NovaSeq 6000 system by Gene Denovo Biotechnology Co. (Guangzhou, China). Briefly, RNA quality was assayed on an Agilent 2100 Bioanalyzer (Agilent Technologies, Palo Alto, CA, USA) and checked via RNase‐free agarose gel electrophoresis. mRNAs were enriched from total RNA via oligo(dT) beads. The enriched mRNAs were subsequently fragmented into short fragments using the fragmentation buffer and reverse transcribed into cDNAs using the NEBNext Ultra RNA Library Prep Kit for Illumina (7530; New England Biolabs). The purified double‐stranded cDNA fragments were end‐repaired, A base was added, and the fragments were ligated to Illumina sequencing adapters. The ligation reaction mixture was purified with AMPure XP beads (1.0×). Ligated fragments were subjected to size selection via agarose gel electrophoresis and amplified via PCR. The resulting cDNA library was subjected to sequencing.

RNA differential expression analysis was performed using DESeq2 software. Genes whose false discovery rate (FDR) was less than 0.05 and whose absolute fold change was ≥ 1.5 were considered differentially expressed genes. ClusterProfiler software was used for all enrichment analyses, including GO enrichment and KEGG (http://www.kegg.jp/) analyses. *p*adj < 0.05 was considered to indicate significant enrichment. GSEA and MsigDB software were used for gene set enrichment analysis to identify whether a set of genes associated with specific GO terms and KEGG pathways significantly differed between the two groups. The RNA sequencing data were deposited to Gene Expression Omnibus data base(GEO) ‐under accession number GSE275892.

### 5.16 Assay of TCGA Datasets

The gene set utilized for the NK inhibitory function or suicide‐related gene (*CISH*, *SOCS2*, *TIGIT*, *CD96*, *CD94*, *NKG2A*, *LAG3*, *TIM3*, *TNFAIP8L2*, *KLRG1*, *KLRB1*, and *CD38*). Analyses of correlation were undertaken using the GEPIA2 Internet website (http://gepia2.cancer‐pku.cn/#index) [28].

### 5.17 Sanger Sequencing and mRNA Stability Assay

Total RNA was extracted by TRIzol reagent and subjected to cDNA synthesis using HiScript III RT SuperMix for qPCR (+gDNA wiper) Kit (R323, Vazyme). The cDNA was used as a template to amplify sequences containing RNA‐seq‐identified ADAR1‐dependent putative editing sites. Individual clones were sequenced using the ABI 3730XL machine in Sangon Biotech. The frequency of different base peaks from the Sanger sequence was quantified using the BEAT program [63]. = mRNA stability assay: NK cells were treated with 5 µg/mL Actinomycin D (ActD, S8964, Selleck) for different times and collected for *CD38* mRNA level detection. The relative mRNA expression at different time points was calculated as the fold change from the baseline.

### 5.18 Tumor Model

For the xenograft tumor model, A375 cells (1 × 10^5^ in 100 µL of PBS) were injected subcutaneously into the right flank of NCG mice. The tumors were allowed to grow for 7 days, and the mice were then treated with PBS or NK‐92 cells at the indicated time points (n = 4 per group). For the NK‐92 cell treatment experiments, NK scramble and NK shADAR1 cells (1 × 10^6^ cells in 100 µL of PBS per mouse) were injected via the tail vein, and all mice were intraperitoneally injected with the human recombinant protein IL‐2 every 2 days to support NK cell survival, as previously reported [36]. To detect the tumor infiltration level of NK cells, NK scramble and NK shADAR1‐2 cells were stained with DIR dye (D12731, Thermo) before i.v. injection following the manufacturer's instrument. The distribution of NK cells in vivo was then scanned with Xenogen IVlS Spectrum Imaging Systems. Tumor burden was monitored every week by measuring the tumor diameter using a Vernier scale. Tumor volume was calculated as V (mm^3^) = 0.5 ×  (length × width^2^). When the tumor volume reached approximately 1000 mm^3^, all the mice were euthanized, and the tumors were collected for analysis.

For the pulmonary metastasis model, 6‐8‐week‐old male mice were inoculated with 1 × 10^5^ B16 cells in 200 µL of PBS via the tail vein. Twenty‐one days after the injection of tumor cells, the mice were sacrificed, and metastatic lung infiltration was evaluated.

### 5.19 NK Cell Infiltration Assay

To study the infiltration potential of ADAR1‐knockdown NK cells into tumor tissue, a 3D protocol from Osswald et al. was adapted with some modifications [64]. In brief, 1 × 10^4^ A375 cells were resuspended in ice‐cold 2×Matrigel (356230, BD), and a 10 µL drop of Matrigel was placed in Costar ultralow attachment round‐bottom 96‐well plates to form a Matrigel spheroid. After 30 mins of incubation at 37°C, 100 µL of DMEM supplemented with 10% FBS was added to each well, and the spheroids were incubated for 48 h. After the medium was replaced with fresh medium, 1 × 10^4^ NK cells stained with CFSE were added to each well and cocultured with spheroids for another 48 h. After removing noninfiltrated NK cells with PBS, images were captured using a microscope (Nikon, Japan). The quantification of fluorescent cells in the spheroids was performed via ImageJ software. Six wells were used for each group.

### 5.20 Statistical Analysis

The data are presented as the mean ± standard error of mean (Mean ± SEM). Statistical analyses were performed using GraphPad Prism version 9.0. Statistical comparisons were made via two‐tailed Student's unpaired t tests between two groups or one‐way analysis of variance (ANOVA) for multigroup comparisons. Correlations between variables were evaluated using the Spearman rank correlation test for data from TCGA, GTEx, or qRT‐PCR. Where indicated, * *p* < 0.05, ** *p* < 0.01, *** *p* < 0.001, and **** *p* < 0.0001 were considered statistically significant.

## Conflicts of Interest

The authors declare no conflicts of interest.

## Supporting information




**Supporting File**: advs73774‐sup‐0001‐SuppMat.docx.

## Data Availability

The data that support the findings of this study are available from the corresponding author upon reasonable request.
