# Supplementary material for: Depletion of the RNA‐Editing Enzyme ADAR1 Invigorates the Antitumor Immunity of NK Cells
Source: Adv Sci (Weinh). 2026 Jan 20;13(29):e17216. doi: 10.1002/advs.202517216 (PMC13205767; doi:10.1002/advs.202517216)
Supplement: Supplementary file 1 — Supporting File: advs73774‐sup‐0001‐SuppMat.docx. [file ADVS-13-e17216-s001.docx]

**Supporting Information**

**Depletion of the RNA-editing enzyme ADAR1 invigorates the antitumor immunity of NK cells**

*Shuhan Chen*, *Di Lu*, *Rukang Liang*, *Weikeng Tan*, *Erming Zhao* , *Sihuang Wu*, *Xintong Li*, *Yulong Song, Miaojian Wan*, *Xiaoyuan Xie**, *Qi Zhang**, *and* *Qiuli Liu**


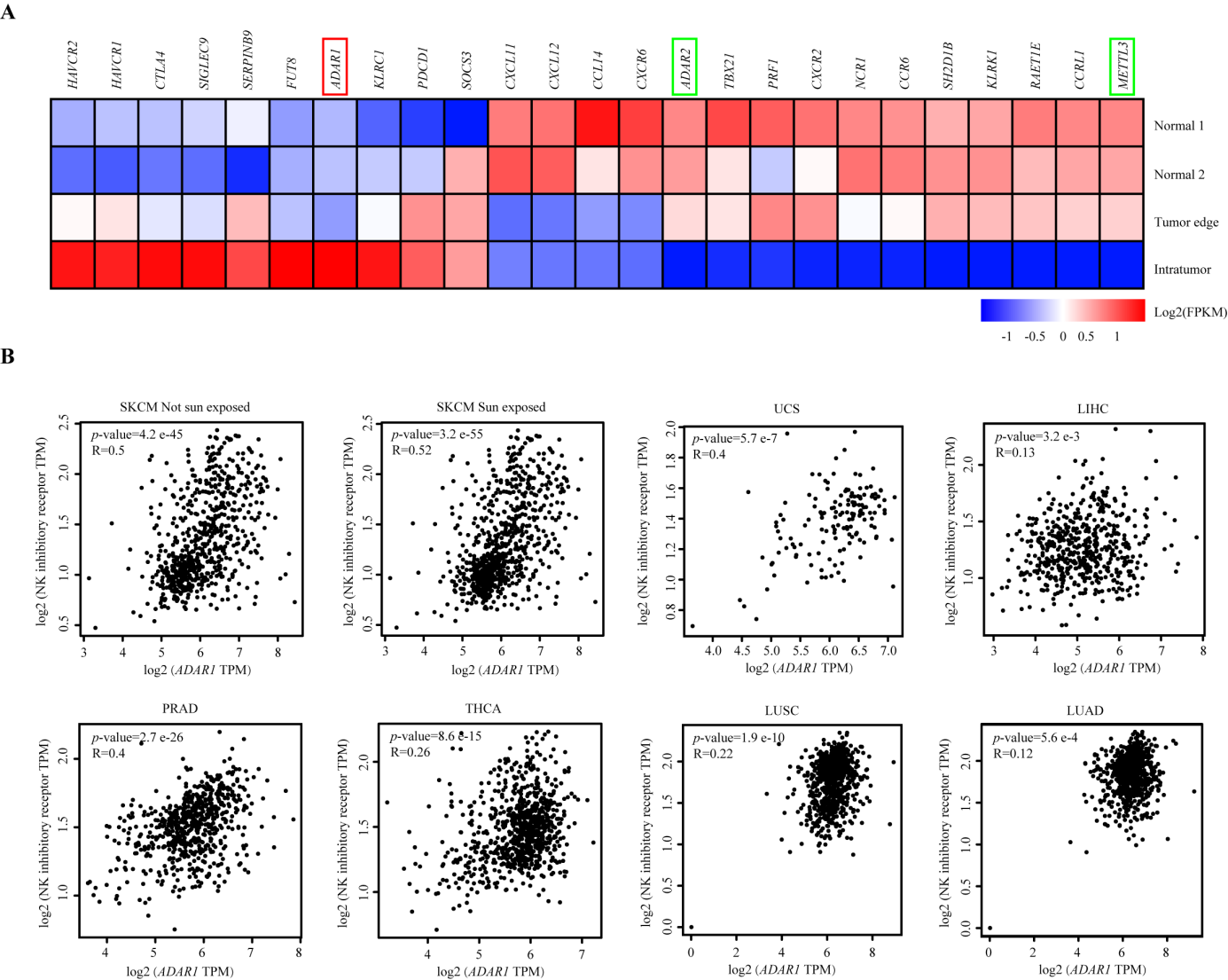


**Figure S1. ADAR1 was positively correlated with inhibitory function of NK cells.** Heatmap showing the NK cells inhibitory function, effector function, and migration-related genes in tumor-infiltrating NK cells compared with those in normal liver NK cells from a published dataset (GSE120123) (A). Correlation between *ADAR1* TPM (transcripts per kilobase of exon model per million mapped reads) and module TPM of NK inhibitory function or suicide-related gene (*CISH*, *SOCS2*, *TIGIT*, *CD96*, *CD94*, *NKG2A*, *LAG3*, *TIM3*, *TNFAIP8L2*, *KLRG1*, *KLRB1*, and *CD38*) in skin cutaneous melanoma (SKCM) tissues, uterine carcinosarcoma (UCS), liver hepatocellular carcinoma (LIHC), prostate adenocarcinoma (PRAD), thyroid carcinoma (THCA), lung squamous cell carcinoma (LUSC), and lung adenocarcinoma (LUAD). Spearman’s correlation coefficient (R) and *p* values are shown (B).


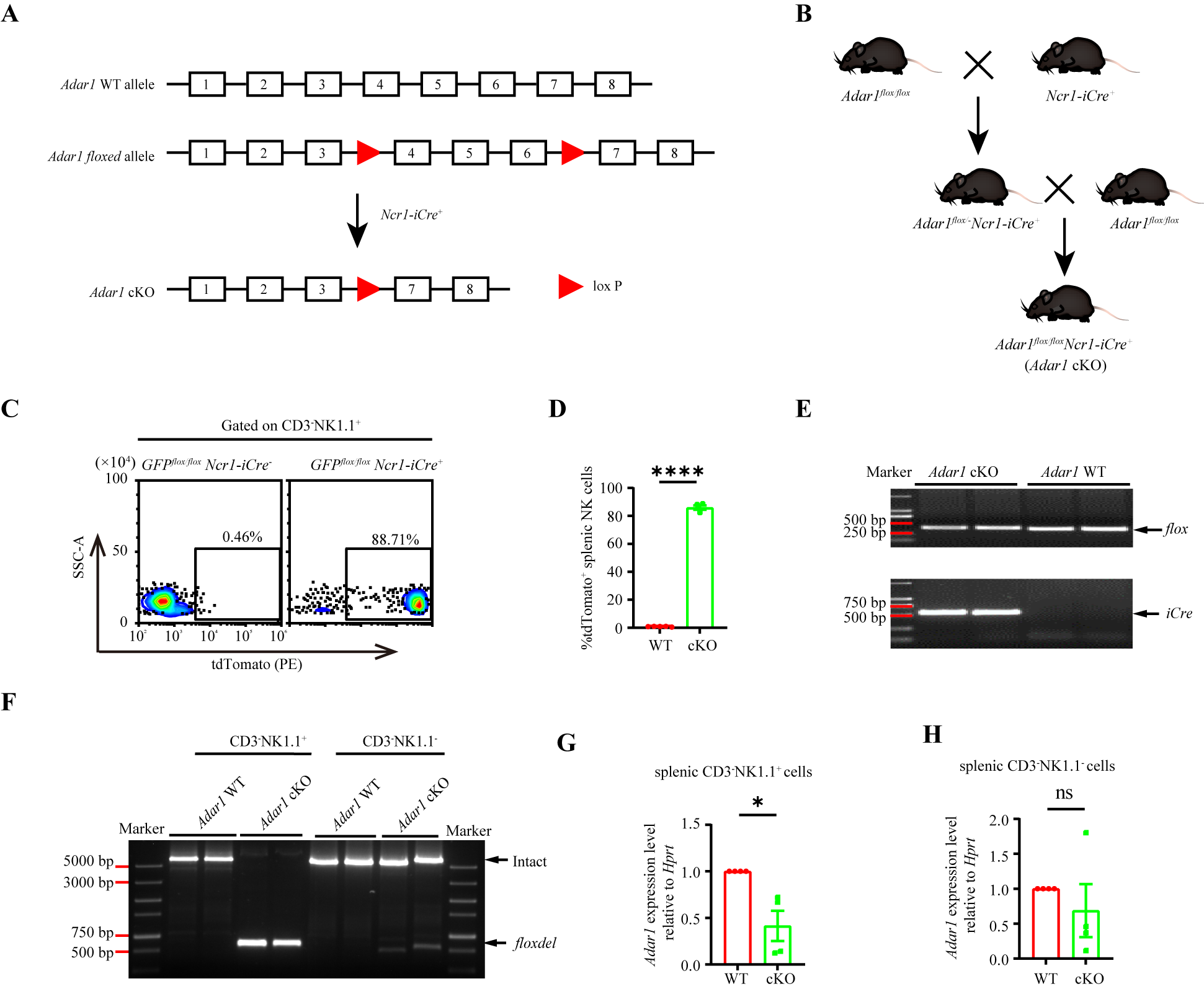


**Figure S2. Flow cytometry analysis the cutting efficacy mediated by *Ncr1-iCre*.** Strategy of *Ncr1-iCre*-mediated *Adar1* knockout mouse construction. *Adar1* *floxed* allele was generated by flanking exons 4 and 6 with loxP sites. NK cell-specific *Adar1* knockout mice (*Adar1* cKO) were obtained by crossing *Adar1* *floxed* mice with *Ncr1-iCre* transgenic mice (A). Schematic diagram of breeding strategy of *Adar1* cKO mice (B). Flow cytometry analysis (C) and statistical graph (D) of the percentage of tdTomato^+^ splenic CD3^-^NK1.1^+^ NK cells derived from 6-week old WT (n=5) and cKO mice (n=4). Genomic PCR for the tail of indicated genotype. The top lane (*flox*) displayed genotyping of homozygous (*Adar1^flox/flox^*) *flox* flanking alleles. The bottom lane (*Ncr1-iCre*) showed the effective insertion of *Ncr1* promoter-driven *iCre* (n=2 for each group) (E). Genomic PCR analysis for CD3^-^NK1.1^-^ and CD3^-^NK1.1^+^ cells isolated from the spleen of mice using magnetic enrichment with indicated genotype (n=2 for each group) (F). qRT-PCR for *Adar1* of CD3^-^NK1.1^+^ (G) and CD3^-^NK1.1^-^ (H) cells isolated from the spleen of mice with indicated genotype (n=4 for each group).


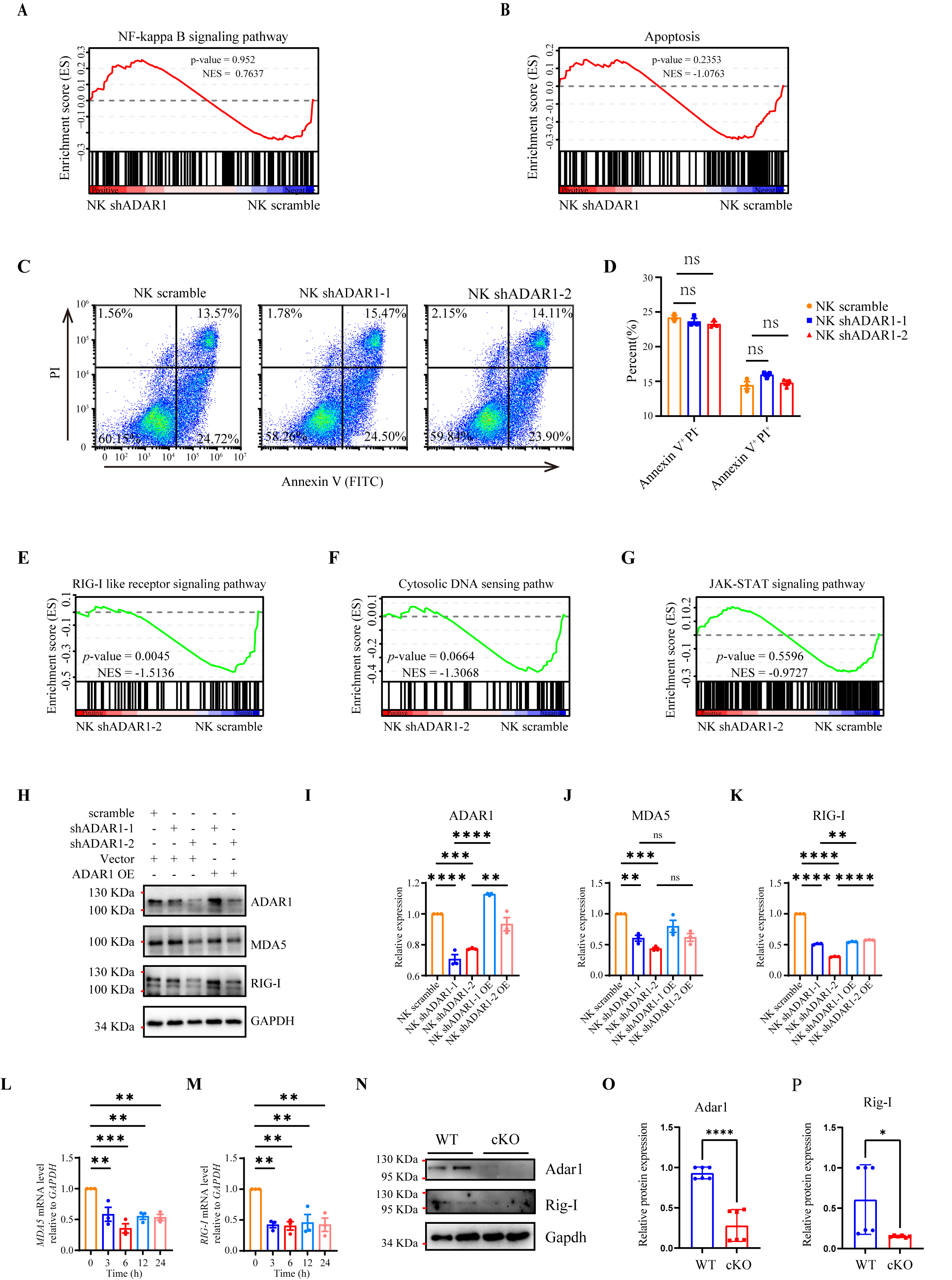


**Figure S3. ADAR1 knockdown had minimal effects on NK-92 cells apoptosis.** GSEA results of NF-κB signaling pathway (A) and apoptosis (B) in NK scramble and NK shADAR1 cells (n=2 for each group). Representative flow cytometry plots (C) and quantification (D) of apoptosis in NK scramble and NK shADAR1 cells (n=3 for each group).GSEA result of RIG-I like receptor signaling pathway (E), cytosolic DNA sensing pathway (F), and JAK-STAT signaling pathway (G) in NK scramble and NK shADAR1 cells. Western blot analysis (H) and statistical graphs of ADAR1 (I), MDA5 (J), and RIG-I (K) in NK scramble and NK shADAR1 cells (n=3 for each group). mRNA level of *MDA5* (L) and *RIG-I* (M) in NK-92 cells treated with 10 μM ADAR1 inhibitor 8-Azaadenosine for different time. Western blot analysis (N) and statistical graphs of Adar1 (O), Rig-I (P) in splenic NK cells derived from *Adar1* WT and cKO mice.

**Supplementary Table S1. Clinical characteristics for patients with melanoma**

| **Patients** | **Age bracket^A^** | **Stage^B^** | **Site of metastasis^C^** | **Treatments** | **Clinical status^D^** | **Tumor size (cm)** |
| --- | --- | --- | --- | --- | --- | --- |
| Patient 1 | Adult | II | NO | Surgery | SD | 1×1 |
| Patient 2 | Adult | III | LN | Surgery, Toripalimab | PD | 2×1.5 |
| Patient 3 | Adult | III | LN | Surgery, Toripalimab | CR | 2×2 |
| Patient 4 | Adult | I | NO | Surgery | SD | 0.2×0.2 |
| Patient 5 | Adult | III | skin | Surgery, Pembrolizumab, Temozolomide | CR | 2.5×2.5 |
| Patient 6 | Adult | I | NO | Surgery | SD | 0.5×0.5 |
| Patient 7 | Adult | II | NO | Surgery, Toripalimab | SD | 1×3 |
| Patient 8 | Adult | II | NO | Surgery | SD | 5×2 |
| Patient 9 | Adult | I | NO | Surgery | SD | 2×5 |
| Patient 10 | Adult | II | NO | Surgery, Pembrolizumab | CR | 10×6 |
| Patient 11 | Adult | II | NO | Surgery | PD | 2×1.5 |

**^A^** Age at diagnosis. **^B^** Stage according to the American Joint Committee on Cancer staging system. **^C^** SD: stable disease; PD: progressive disease; CR: chronic response. **^D^** LN: lymph node

**Supplementary Table S2. Primers used for genotyping and CD38 editing site identification**

| **Name** | **Sequences (5’→3’)** |
| --- | --- |
| 5’ arm *Ncr1-icre* F1 | GGTTGCAGACTGAGCAGTTGATGG |
| 5’ arm *Ncr1-icre* R1 | TGCACACAGACAGGAGCATCTTCC |
| *Ncr1-cre* R2 | ACCAGAAGCAGGTATCCCAGGTGA |
| 5’ arm *Adar1-flox* F1 | ACAGTCTTCTGGGATAGCACTCCTG |
| 5’ arm *Adar1- flox* R1 | ATATCAGAACCAGCTTCTCTGGGC |
| 3’ arm *Adar1- flox* F1 | TCTGAGGCGGAAAGAACCAG |
| 3’ arm *Adar1- flox* R1 | ATGGCCGACTATGATACATCTGC |
| CD38-15852061-F | GTGGGGATATGTTCTGAGAAAT |
| CD38-15852061-R | TTGTATCACAGTGTGCAGACAT |

**Supplementary Table S3. Sequence of shRNA for ADAR1**

| **Name** | **Sequences** |
| --- | --- |
| shADAR1-1F | GCACTGGCAGTCTCCGGGTGT |
| shADAR1-1R | ACACCCGGAGACTGCCAGTGC |
| shADAR1-2F | GCCGTGTCCCGAGGAAGTGCA |
| shADAR1-2R | TGCACTTCCTCGGGACACGGC |
| Scramble-F | AATTCTCCGAACGTGTCACGT |
| Scramble-R | ACGTGAAACGTTCGGAGAATT |

**Supplementary Table S4. Antibodies used in this study**

| **Antibodies** | **Source** | **Country** | **Catalog No.** |
| --- | --- | --- | --- |
| V450-conjugated anti-CD3 antibody | BD | USA | 560365 |
| FITC-conjugated anti-CD3 antibody | Biolegend | USA | 300306 |
| PE-Cy7-conjugated anti-CD56 antibody | Biolegend | USA | 362510 |
| APC-conjugated anti-CD56 antibody | Biolegend | USA | 362504 |
| PE-conjugated anti-CD38 antibody | BD | USA | 555460 |
| Brilliant Violet 421-conjugated anti-CD38 antibody | Biolegend | USA | 102732 |
| Alexa Fluor™ 488-conjugated goat anti-mouse IgG | Thermo | USA | A-11001 |
| Alexa Fluor™ 488-conjugated goat anti-rabbit IgG | Thermo | USA | A-11008 |
| PE/Cyanine7 anti-mouse/human CD11b Antibody | Biolegend | USA | 101216 |
| FITC anti-mouse/rat/human CD27 Antibody | Biolegend | USA | 124207 |
| PE anti-mouse CD244.2 (2B4 B6 Alloantigen) Antibody | Biolegend | USA | 133507 |
| PE anti-mouse CD335 (NKp46) Antibody | Biolegend | USA | 137604 |
| PerCP/Cyanine5.5 anti-mouse CD226 (DNAM-1) Antibody | Biolegend | USA | 1128813 |
| APC anti-mouse CD314 (NKG2D) Antibody | Biolegend | USA | 130211 |
| APC anti-mouse CD178 (FasL) Antibody | Biolegend | USA | 106610 |
| PE anti-mouse CD69 Antibody | Biolegend | USA | 104508 |
| PerCP/Cyanine5.5 anti-T-bet Antibody | Biolegend | USA | 644805 |
| Anti-ADAR1 antibody | Santa Cruz Biotech | USA | sc-73408 |
| Anti-CD56 antibody | Servicebio | Chnia | GB12041 |
| Anti-Ki-67 antibody | Abcam | England | ab15580 |
| Anti-NCR1 antibody (mouse) | Abcam | England | ab283505 |
| Anti-NCR1 antibody (human) | Abcam | England | Ab224703 |
| Anti-GAPDH antibody | Cell Signaling Technology | USA | 5174S |
| Anti-MDA5 antibody | Abcam | England | ab283311 |
| Anti-RIG1 antibody | Cell Signaling Technology | USA | 3743S |
| Anti-mouse IgG, HRP-linked antibody | Cell Signaling Technology | USA | 7076 |
| Anti-rabbit IgG, HRP-linked antibody | Cell Signaling Technology | USA | 7074 |

**Supplementary Table S5. Primers used for RT-qPCR**

| Name | Sequences |
| --- | --- |
| Human *ADAR1* total-RT-F | TGCTGCTGAATTCAAGTTGG |
| Human *ADAR1* total-RT-R | TCGTTCTCCCCAATCAAGAC |
| Human *HPRT*-RT-F | CGTCTTGCTCGAGATGTGATG |
| Human *HPRT*-RT-R | TTTATAGCCCCCCTTGAGCAC |
| Human *CD38*-RT-F | TGGGAACTCAGACCGTACCT |
| Human *CD38*-RT-R | TGCTGCAGTCCTTTCTCCAG |
| Human *MDA5*-RT-F | GAGCAACTTCTTTCAACCACAG |
| Human *MDA5*-RT-R | CACTTCCTTCTGCCAAACTTG |
| Human *RIG1*-RT-F | GGTGTTCCAGATGCCAGACA |
| Human *RIG1*-RT-R | TCATCGAATCCTGCTGCTCG |
| Human *GAPDH*-RT-F | CAAGGTCATCCATGACAACTTTG |
| Human *GAPDH*-RT-R | GTCCACCACCCTGTTGCTGTAG |
| Mouse *Adar1* total-RT-F | TGAGCATAGCAAGTGGAGATACC |
| Mouse *Adar1* total-RT-R | GCCGCCCTTTGAGAAACTCT |
| Mouse *Hprt*-RT-F | TCAGTCAACGGGGGACATAAA |
| Mouse *Hprt*-RT-R | GGGGCTGTACTGCTTAACCAG |
| Mouse *Cd38*-RT-F | ACCACGAAGCACTTTTCTGAC |
| Mouse *Cd38*-RT-R | GGCGTAGTCTTCTCTTGTGATG |
| Mouse *Gapdh*-RT-F | AATGGATTTGGACGCATTGGT |
| Mouse *Gapdh* -RT-R | TTTGCACTGGTACGTGTTGAT |

**Supplementary Table S6. Reagents used in this study**

| **Reagents** | **Source** | **Country** | **Identifier** |
| --- | --- | --- | --- |
| CFSE | Invitrogen | USA | C34554 |
| Fixation/Permeabilization Kit | BD | USA | 554714 |
| 0.45 μm Nonpyrogenic Filter | JET BlOFIL | USA | FPV403150 |
| ChamQ SYBR Color qPCR Master Mix | Vazyme | China | Q421 |
| HiScript III RT SuperMix for qPCR (+gDNA wiper) Kit | Vazyme | China | R323 |
| LDH Cytotoxicity Assay | DOJINDO | Japan | CK12 |
| FITC Annexin V Apoptosis Detection Kit | BD | USA | 556547 |
| human NK Cell Serum-free Culture Kit | YOCON | China | NC0102.F, NC0102, and AN0104 |
| Matrigel | BD | USA | 356230 |
| Immonilon ECL Ultra Western HRP Substrate | Millipore | USA | WBULS0500 |
| Puromycin | MCE | China | HY-K1057 |
| Polybrene | MCE | China | HY-112735 |
| Polyethylenimine (PEI) | MCE | China | HY-K2014 |
| Penicillin/Streptomycin | KeyGEN Biotech | China | KGY0023 |
| DMEM-High Glucose Medium | Thermo Scientific | USA | C11995500BT |
| Fetal Bovine Serum | ExCell | China | FSP100 |
| RPMI 1640 Medium | GIBCO | USA | 11875119 |
| β-Mercaptoethanol | GIBCO | USA | 21985023 |
| Recombinant Human IL-2 | Quangang Pharmaceutical | China | S20020004 |
| Actinomycin D | Selleck | China | S8964 |
| TRIzol | Invitrogen | USA | 15596026 |
